# Kinetic and structural studies of *Trypanosoma* and *Leishmania* phosphofructokinases show evolutionary divergence and identify AMP as a switch regulating glycolysis *versus* gluconeogenesis

**DOI:** 10.1111/febs.15177

**Published:** 2020-01-08

**Authors:** Peter M. Fernandes, James Kinkead, Iain W. McNae, Monserrat Vásquez‐Valdivieso, Martin A. Wear, Paul A. M. Michels, Malcolm D. Walkinshaw

**Affiliations:** ^1^ Centre for Translational and Chemical Biology School of Biological Sciences The University of Edinburgh Edinburgh UK

**Keywords:** allostery, glycolysis, *Leishmania*, phosphofructokinase, *Trypanosoma*

## Abstract

Trypanosomatids possess glycosome organelles that contain much of the glycolytic machinery, including phosphofructokinase (PFK). We present kinetic and structural data for PFK from three human pathogenic trypanosomatids, illustrating intriguing differences that may reflect evolutionary adaptations to differing ecological niches. The activity of *Leishmania* PFK – to a much larger extent than *Trypanosoma* PFK – is reliant on AMP for activity regulation, with 1 mm AMP increasing the *L. infantum* PFK (LiPFK) kcat/K_0.5_^F6P^ value by 10‐fold, compared to only a 1.3‐ and 1.4‐fold increase for *T. cruzi* and *T. brucei* PFK, respectively. We also show that *Leishmania* PFK melts at a significantly lower (> 15 °C) temperature than *Trypanosoma* PFKs and that addition of either AMP or ATP results in a marked stabilization of the protein. Sequence comparisons of *Trypanosoma* spp. and *Leishmania* spp. show that divergence of the two genera involved amino acid substitutions that occur in the enzyme’s ‘reaching arms’ and ‘embracing arms’ that determine tetramer stability. The dramatic effects of AMP on *Leishmania* activity compared with the *Trypanosoma* PFKs may be explained by differences between the T‐to‐R equilibria for the two families, with the low‐melting *Leishmania* PFK favouring the flexible inactive T‐state in the absence of AMP. Sequence comparisons along with the enzymatic and structural data presented here also suggest there was a loss of AMP‐dependent regulation in *Trypanosoma* species rather than gain of this characteristic in *Leishmania* species and that AMP acts as a key regulator in *Leishmania* governing the balance between glycolysis and gluconeogenesis.

## Graphical abstract

All trypanosomatid apo phosphofructokinases (PFKs) have low affinity for fructose 6‐phosphate (F6P). ATP partially primes the enzyme, which can then bind F6P with medium affinity and permit avid binding of AMP. In the fully primed AMP‐bound state, PFK has a higher affinity for F6P. AMP has a significantly greater effect on *Leishmania* PFK than on *Trypanosoma cruzi* or *T. brucei* PFK; this is rationalized by the occupation of different ecological niches.

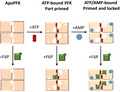

**Abbreviations**
AMP: adenosine monophosphate
ATP: adenosine triphosphate
ATP‐PFK: ATP‐dependent phosphofructokinase (EC 2.7.1.11)
F16BP: fructose 1,6‐bisphosphate
F26BP: fructose 2,6‐bisphosphate
F6P: fructose 6‐phosphate
FBPase: fructose‐1,6‐bisphosphatase (EC 3.1.3.11)
LiPFK: *Leishmania infantum* phosphofructokinase
PFK: phosphofructokinase (EC 2.7.1.11)
PPi‐PFK: PPi‐dependent phosphofructokinase (EC 2.7.1.90)
RMSD: root‐mean‐square deviation of atomic position
RU: response units
SPR: surface plasmon resonance
TbPFK: *Trypanosoma brucei* phosphofructokinase
TcPFK: *Trypanosoma cruzi* phosphofructokinase

## Introduction

The Trypanosomatida order is comprised of exclusively parasitic species, including the medically relevant genera *Leishmania* and *Trypanosoma*. Trypanosomatids are unique in possessing glycosomes, specialized membrane‐bounded metabolic organelles related to peroxisomes 1, 2, 3. Each parasite contains multiple glycosomes, with bloodstream‐form *T. brucei* possessing approximately 65 glycosomes per cell, each with a diameter about 0.3 µm, comprising 4–5% of the total cell volume 4. The majority of the glycolytic and gluconeogenic pathway enzymes are sequestered in the glycosomes along with some other metabolic enzymes. The functions of the glycosomes, and the possible evolutionary drivers for its origin, have been the subject of much debate 2, 3. The singular nature of trypanosomatid metabolism caused these pathogens – particularly *T. brucei* – to be extensively studied 5.

Glycosomes contain phosphofructokinase (PFK), which catalyses the third step of glycolysis: the phosphorylation of fructose 6‐phosphate (F6P) to fructose 1,6‐bisphosphate (F16BP) via the conversion of ATP to ADP. This reaction involves a large negative free energy change and is the first committed step of glycolysis. Trypanosomatid PFKs, notably the enzyme of *T. brucei* (TbPFK), have been studied in great detail 6, 7, 8, 9, 10, 11, 12, 13, 14, 15, 16, 17, 18, 19. They have high overall amino acid sequence identities (Table 1), with complete conservation of substrate binding sites for both ATP (first described in TbPFK crystal structure 6, 19) and F6P (with the F6P site inferred by comparison with the *Escherichia coli* crystal structure 20), as shown in Fig. S1. Crystal structures show TbPFK is a tetramer of four 53‐kDa subunits 6, 19. However, there are low amino acid sequence identities of 23–26% between human PFK isoforms and the trypanosomatid PFKs 7. This low identity is attributed to a complex evolutionary trajectory. Phylogenetic analysis demonstrated that trypanosomatid PFKs are evolutionarily related to inorganic pyrophosphate‐dependent PFKs (PPi‐PFK; EC2.7.1.90), while all known metazoan PFKs belong to the ATP‐dependent PFKs (ATP‐PFK; EC2.7.1.11). These two groups are homologous, yet have a very distant common ancestral enzyme 7. Nonetheless, trypanosomatid PFKs exclusively use ATP as a phospho‐donor, indicating that they must have undergone a change in phospho‐donor specificity during their evolution 6, 7. This ancestry also explains differences in the active site when comparing the *T. brucei* PFK crystal structure on the one hand with authentic PPi‐PFKs and on the other hand with ATP‐PFKs 6, 19. Furthermore, the activity of trypanosomatid PFKs is not regulated by any of the common regulators of well‐studied bacterial, yeast, plant and mammalian ATP‐PFKs 8, 9, 10, 11, 12, 13, 14, 15, 16, 17, 18, 20, 21. This may be due in part to the different ancestry, but also to adaptation to the unique compartmentation of trypanosomatid PFKs within glycosomes 1, 2, 3.

**Table 1:** Trypanosomatid PFK amino acid sequences share high percentage sequence identities. Amino acid sequences were retrieved from www.uniprot.org; alignments made using clustal omega 2.1^a^.

	*L. mexicana*	*L. infantum*	*L. donovani*	*T. brucei*	*T. cruzi*
*L. mexicana*	100%	98%	97%	70%	71%
*L. infantum*		100%	100%	70%	72%
*L. donovani*			100%	70%	72%
*T. brucei*				100%	77%
*T. cruzi*					100%

a http://www.ebi.ac.uk/Tools/msa/clustalo/.

Many Trypanosomatida are monoxenous (one host) insect parasites, with *Leishmania*, *Phytomonas* and *Trypanosoma* being the only dixenous (two hosts) genera within the order. Phylogenetic trees indicate divergence between the *Leishmania* and *Trypanosoma* genera occurred around 120 million years ago, with a subsequent split occurring between salivarian and stercorarian *Trypanosoma* species, represented by the African *T. brucei* and American *T. cruzi*, respectively, approximately 100 million years ago 22, 23. *T. brucei* lives in extracellular fluids only, with no intracellular stage; in the human host, *T. brucei* is highly glucose‐dependent, exhibiting a very high glycolytic flux 2, though not in the tsetse fly stages, where proline and other amino acids are the main sources for ATP production 24. *T. cruzi* has an intracellular stage as amastigotes in the cytosol of a variety of cell types, and conflicting information exists about the use of glucose by amastigotes; its intracellular concentration in host cells is usually scarce, and the parasite glucose transporter was shown to be downregulated 12. However, a recent publication reported that restriction of extracellular glucose impaired amastigote proliferation and that intracellular *T*. *cruzi* amastigotes capitalize on the host metabolic response to parasite infection by increasing glucose uptake to fuel their own metabolism and replication in the host cytosol 25. In contrast, sugars are probably also limited for the insect epimastigote forms 26. *Leishmania* spp. are even more specialized, only transforming from the extracellular promastigotes in the sandfly into amastigotes inside the phagolysosome of mononuclear phagocytes. It was previously assumed that glycolysis was unimportant in this low‐sugar environment, but it has recently been shown that *L. mexicana* is reliant on glycolysis in activated macrophages 27. The concentrations of glucose and other saccharides in each of these environments are very different, and trypanosomatid PFKs will have evolved different kinetic properties to adapt to their respective habitats.

Here, we present a comparison of the enzyme kinetic and structural differences of heterologously expressed, fully purified recombinant PFKs of three disease‐causing trypanosomatid species. No data have previously been published on *Leishmania infantum* PFK (LiPFK), although data are available on other, closely related (Table 1) leishmanial PFKs 8, 9. Reports of the kinetic properties of *T. cruzi* PFK (TcPFK) and TbPFK have been previously published (references stated in Table 2), though these have never been directly compared to each other, or with any leishmanial PFK 8, 9, 10, 11, 12, 13, 14, 15, 16, 17, 18. AMP is a long‐recognized allosteric activator of TbPFK 16, and the X‐ray structure of TbPFK in complex with AMP presented here provides new insight into the reaction ordering and allosteric mechanisms of these enzymes and, together with the kinetic data, offers an intriguing perspective on the evolution of metabolic regulation as adaptation to the respective environments of these parasites.

**Table 2:** Biochemical parameters for trypanosomatid PFKs incorporating all previous studies and the values obtained from this work.

Species	Source	K_0.5_^ATP^ (mm)	K_0.5_^F6P^ (mm)	*V*_max_ µmol·min^−1^·mg^−1^	Conditions	Reference
*T. cruzi*	Partially purified parasite	0.04	1.31 (0.37 if 0.5 mm AMP)	0.4	1 mm ATP 1 mm F6P	11, 18
	Recombinant (*E. coli*)	0.0125	–	–	1 mm F6P	12, 19
	Purified parasite	0.025	2.8	0.07	0.5 mm ATP 2 mm F6P	13, 20
	Whole parasite homogenate	–	0.29 (epimastigotes) 0.15 (metacyclics)	40.3	1 mm ATP	14, 21
	Partially purified parasite	1.9 (0.05 if 3 mm AMP)	1.75 (0.8 if 0.1 mm AMP)	–	Not stated	15, 22
	Recombinant (*E. coli*)	0.095	0.31	0.068	3 mm F6P 1.5 mm ATP	This work
*T. brucei*	Purified parasite	0.065	0.999	–	5 mm F6P 1 mm ATP	16, 23
	Recombinant (*E. coli*)	0.144	1.15	0.045	0.5 mm ATP 1.6 mm AMP	17, 24
	Purified parasite	0.026	0.82	0.267	2 mm ATP	18, 25
	Recombinant (*E. coli*)	0.050	0.37	0.045	3 mm F6P 1.5 mm ATP	This work
*L. mexicana*	Whole parasite homogenate	–	–	0.138 (promastigotes) 0.089 (amastigotes)	0.4 mm ATP 3.3 mm F6P	10, 17
*L. donovani*	Recombinant (*E. coli*)	–	3.6 (0.157 if 1.5 mm AMP)	Not stated	1 mm ATP	9, 16
	Whole parasite homogenate	0.08	10 (0.5 if 3 mm AMP)	0.29 0.43 with 3 mm AMP	Not stated	8, 15
*L. infantum*	Recombinant (*E. coli*)	0.050	2.89	0.014	3 mm F6P 1.5 mm ATP	This work

## Results

### PFK activity differs between trypanosomatid species

**Figure 1:**
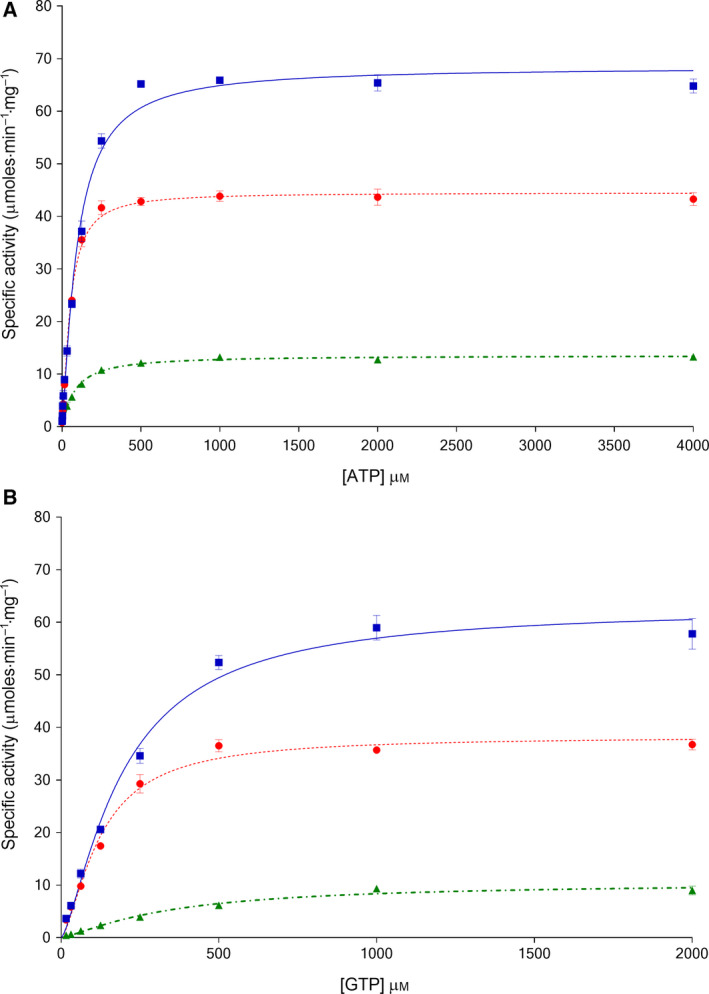
Kinetic plots of *Trypanosoma cruzi* PFK (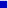, solid blue line), *Trypanosoma brucei* PFK (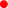, dotted red line) and *Leishmania infantum* PFK (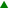, dot‐dashed green line) with respect to ATP titrations (A) and GTP titrations (B) show different kinetic properties for each enzyme. Data were fitted with sigmoidal curves given by the equation for a cooperative (allosteric) model (see the Materials and methods) (*n* = 3; error bars represent standard deviations; 3 mm F6P).

**Figure 2:**
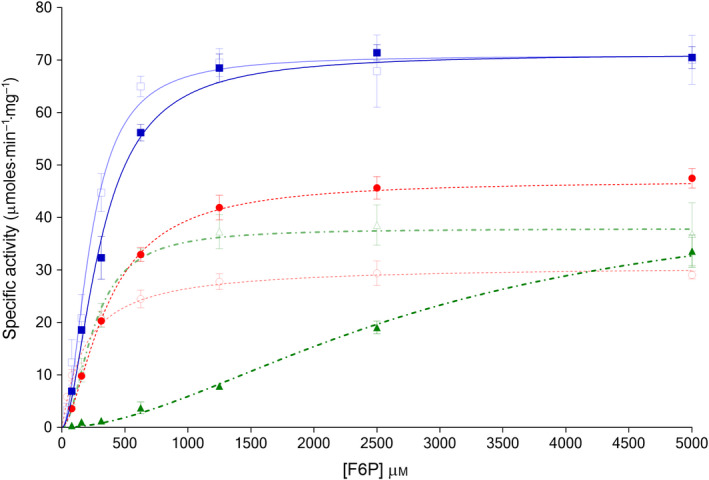
Kinetic plots with respect to fructose 6‐phosphate titrations for *Trypanosoma cruzi* PFK (TcPFK) (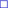, blue line), *Trypanosoma brucei* PFK (TbPFK) (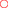, dotted red line) and *Leishmania infantum* PFK (LiPFK) (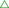, dot‐dashed green line) show different kinetic properties for each enzyme. Effects of 1 mm AMP, shown alongside for TcPFK (□, pale blue line), YbPFK (○, dotted red line) and LiPFK (△, dot‐dashed green line) show disproportionate activation for LiPFK. Data were fitted with a sigmoidal curve given by the equation for a cooperative (allosteric) model (*n* = 3; error bars represent standard deviations; 1.5 mm ATP).

Values of kinetic parameters of all three trypanosomatid PFKs were determined for ATP and F6P, with and without AMP (Figs 1 and 2, Table 3). TcPFK has a lower affinity for ATP (K_0.5_^ATP^ 94.6 μm) compared to other trypanosomatid PFKs, with TbPFK and LiPFK having similar values (K_0.5_^ATP^ 50.4 and 49.6 μm, respectively). In contrast, TcPFK and TbPFK have similar affinities for F6P (K_0.5_^F6P^ 310 and 365 μm, respectively), but LiPFK has a significantly lower affinity, at almost an order of magnitude less (K_0.5_^F6P^ 2886 μm). Comparison of the resulting fits with *F*‐tests (data not shown) indicates that ATP binds with a small degree of cooperativity for TcPFK, TbPFK and LiPFK (h values 1.24, 1.36 and 1.10, respectively), in contrast to previously published literature suggesting that the ATP binding curve is hyperbolic rather than sigmoidal 18. F6P binds in a more cooperative manner (h values 1.78, 1.64 and 1.78, respectively), in keeping with previous studies 8, 11, 18. These results show that cooperativity of binding for PFK substrates does not differ greatly between trypanosomatid species (Table 3).

**Table 3:** Kinetic parameters for TcPFK, TbPFK and LiPFK, for main substrates (F6P, ATP) and accessory substrates (GTP), with and without AMP. Data obtained from minimum *n* = 3; values are mean averages with standard deviations, and ND indicates not done.

AMP	TcPFK		TbPFK		LiPFK	
	0 mm	1 mm	0 mm	1 mm	0 mm	1 mm
F6P						
*V*_max_^F6P^ (μmol·min^−1^·mg^−1^)	71.3 (1.1)	70.9 (1.7)	47.1 (0.7)	30.9 (1.0)	45.1 (2.9)	37.9 (1.3)
K_0.5_^F6P^ (μm)	310 (14)	227 (15)	365 (15)	182 (20)	2886 (310)	253 (25)
k_cat_^F6P^ (s^−1^)	66.4 (1.0)	66.1 (1.6)	43.8 (0.6)	28.7 (1.0)	42.4 (2.7)	35.6 (1.2)
k_cat_^F6P^/K_0.5_^F6P^ (s^−1^·μm ^−1^)	0.21	0.29	0.12	0.16	0.015	0.14
h (F6P)	1.78 (0.13)	1.90 (0.20)	1.64 (0.09)	1.06 (0.11)	1.78 (0.22)	1.91 (0.32)
ATP						
*V*_max_^ATP^ (μmol·min^−1^·mg^−1^)	68.4 (1.0)	64.8 (1.9)	44.5 (1.4)	27.7 (0.6)	13.6 (0.2)	14.1 (0.2)
K_0.5_^ATP^ (μm)	94.6 (4.9)	65.7 (6.1)	50.4 (1.8)	36.9 (2.6)	49.6 (2.8)	51.1 (2.2)
k_cat_^ATP^ (s^−1^)	63.7 (1.02)	60.41 (1.75)	41.4 (1.26)	25.7 (0.52)	12.8 (0.21)	13.2 (0.17)
k_cat_^ATP^/K_0.5_^ATP^ (s^−1^·μm ^−1^)	0.67	0.92	0.82	0.70	0.26	0.26
h (ATP)	1.24 (0.06)	1.39 (0.17)	1.36 (0.06)	1.44 (0.16)	1.10 (0.07)	1.08 (0.06)
GTP						
*V*_max_^GTP^ (μmol·min^−1^·mg^−1^)	63.0 (1.8)	ND	38.3 (0.9)	ND	10.5 (0.7)	ND
K_0.5_^GTP^ (μm)	195 (14)	ND	123 (8)	ND	342 (50)	ND
k_cat_^GTP^ (s^−1^)	58.7 (1.7)	ND	35.6 (0.84)	ND	9.9 (0.62)	ND
k_cat_^GTP^/K_0.5_^GTP^ (s^−1^·μm ^−1^)	0.30	ND	0.29	ND	0.03	ND
h (GTP)	1.38 (0.1)	ND	1.5 (0.12)	ND	1.27 (0.14)	ND

### GTP is an alternative nucleotide substrate

Previous studies have shown that GTP is a possible alternative phospho‐donor for TbPFK 16 and bacterial PFKs 28, though not for LiPFK 8. The data presented in Fig. 1 and Table 3 confirm that GTP is an alternative phospho‐donor for TcPFK, TbPFK and LiPFK (K_0.5_^GTP^ 195, 123 and 342 μm, respectively). An interesting additional observation from running this assay using human muscle pyruvate kinase (PYK‐M) as one of the linker enzymes (see the Materials and methods) is that PYK‐M can use GDP as a substrate; no ADP was present in the GTP experiments and PYK‐M was able to use GDP to convert PEP into pyruvate. This observation is in line with the historical and little‐referenced observation from Hohnadel and Cooper 29.

The physiological relevance of trypanosomatid PFKs using GTP as a phospho‐donor is uncertain but probably low. There are several indications that trypanosomatid glycolysis is generally dependent on ATP, with other nucleoside triphosphates being of much lesser importance. While the concentrations of GDP and GTP have not been directly measured within glycosomes, total cellular concentrations of GTP are significantly lower than ATP, with a calculated GTP/ATP ratio of 0.4 30. Furthermore, ATP‐dependent enzymes in glycosomes have been experimentally detected or indirectly identified through possession of glycosome‐targeting motifs, but no glycosomal GTP‐dependent enzymes have been reported. A third point arguing against the physiological relevance of GTP is that there must be a mechanism within the glycosomes for rephosphorylating the resulting GDP for reuse; the only known glycosomal enzyme that can generate nucleoside triphosphates at the high rates required is phosphoglycerate kinase, which is highly specific for ADP and cannot use other nucleoside diphosphates including GDP 31.

### AMP is the sole known effector of trypanosomatid PFKs

AMP is a known allosteric activator of all trypanosomatid PFKs 8, 11, 18. Values of kinetic parameters for F6P and ATP were determined for all three trypanosomatid PFKs in the presence and absence of 1 mm AMP, as shown in Fig. 2 and Table 3. The addition of AMP increases the affinity of TcPFK and TbPFK for ATP, with respective reductions in K_0.5_ values of about 30%; no change was seen for LiPFK. Reductions in *V*_max_ values were observed for TbPFK and, to a lesser degree, for TcPFK. The reduction in *V*_max_ for both ATP and F6P (particularly for TbPFK) with a concomitant reduction in K_0.5_ is consistent with AMP acting as an ‘uncompetitive inhibitor’ that only forms an enzymatically productive complex with PFK after the substrate (ATP) has bound (see results below on Reaction Order). No significant changes in Hill coefficient values were demonstrated.

The effects of AMP on F6P binding are more marked and are observed in all three trypanosomatid PFKs. The affinity for F6P of TcPFK and TbPFK increases significantly in the presence of AMP, with respective reductions in K_0.5_ by 27% and 50%. LiPFK is a special case, with a massive increase in affinity for F6P shown by a reduction in K_0.5_ by 91% and a much improved kcat/K_0.5_ value, now similar to the other trypanosomatid PFKs (TcPFK 0.29 s^−1^·μm ^−1^; TbPFK 0.16 s^−1^·μm ^−1^; LiPFK 0.14 s^−1^·μm ^−1^). Measurements of cooperativity in the presence of AMP showed a significant change to a hyperbolic binding model for F6P binding to TbPFK (Hill coefficient reducing to 1.06 from 1.64). The overall cellular AMP concentration in bloodstream‐form *T. brucei* has been estimated to vary between 0.25 and 2.2 mm during its growth 30 and suggests that the activation effects shown in our kinetic studies are biologically relevant.

Addition of 0.5 mm GMP had a similar effect to AMP on both TcPFK and TbPFK (kcat/K_0.5_^F6P^ increases by 33% and 43%, respectively) (data not shown). The biological relevance of activation by GMP is unknown. Screening of several common metabolites at a concentration of 1 mm using fixed substrate concentrations (corresponding to the respective K_0.75_^F6P^ and K_0.75_^ATP^ values) did not show any significant effects for any trypanosomatid PFK (data not shown). The metabolites included ADP, α‐ketoglutarate, cyclic AMP, citrate, fumarate, fructose 2,6‐bisphosphate, glucose 6‐phosphate, GDP, malate, oxaloacetate, PEP, PPi, pyruvate and succinate. TcPFK was used as a screen to investigate the effect of amino acids. All 20 naturally occurring amino acids were tested at 1 mm concentrations, with none demonstrating significant effects on TcPFK activity (data not shown).

### Reaction order influences kinetic parameters

Altering the sequence of addition of reaction components for TbPFK results in marked effects on kinetic properties. In a separate series of experiments to examine this effect, we show that when ATP is incubated with His‐tagged TbPFK for 15 min and the reaction is started by adding F6P, the K_0.5_^F6P^ is 596 ± 19 µm. However, reversing the order and incubating TbPFK with F6P for 15 min before initiating the reaction with ATP give a considerably larger K_0.5_^F6P^ of 1220 ± 97 µm, suggesting that ATP binds first and somehow primes the F6P binding site. The *V*_max_ of the enzyme is unaffected by the reaction order, remaining at ~ 50 µmol·min^−1^·mg^−1^ for both experiments. These results are consistent with the surface plasmon resonance studies, which could not detect F6P binding unless ATP was present (Table 4).

**Table 4:** Steady‐state SPR affinities for key ligands for each trypanosomatid PFK. (*N* = 3; values are mean averages with standard errors; surface densities: TbPFK = 3200 RU; TcPFK = 3100 RU; LiPFK = 1400 RU).

	TcPFK	TbPFK	LiPFK
AMP			
K_d_^AMP^ (mm)	1.04 (0.30)	0.47 (0.02)	2.3 (0.20)
K_d_^AMP^ + 1 mm ATP (mm)	1.69 (0.10)	1.93 (0.30)	12.2 (4.80)
ADP			
K_d_^ADP^ (mm)	0.73 (0.10)	0.56 (0.08)	0.16 (0.02)
K_d_^ADP^ + 1 mm ATP (mm)	2.13 (0.32)	1.51 (0.11)	0.86 (0.13)
ATP			
K_d_^ATP^ (µm)	35.4 (5.6)	15.8 (2.3)	16.8 (2.1)
K_d_^ATP^ + 1 mm AMP (µm)	36.7 (3.3)	15.1 (1.7)	19.3 (1.1)
F6P			
K_d_^F6P^ (mm)	No binding	No binding	No binding
K_d_^F6P^ + 1 mm ATP (mm)	Weak binding	Weak binding	Weak binding

The order of binding also seems to play a role in AMP effector mechanism and binding. Enzyme activity only appears to be significantly enhanced by AMP if it is added after the enzyme is incubated with ATP. Incubating AMP (at a fixed concentration of 0.5 mm) with PFK for 15 min at room temperature and then adding ATP followed immediately by F6P to start the reaction give a specificity constant (kcat/K_0.5_^F6P^) of 0.07 s^−1^·μm ^−1^ and K_0.5_^F6P^ of 394 ± 19 µm. Reversing the order and incubating PFK first with ATP for 15 min and then adding AMP followed by F6P give an enhanced specificity constant (kcat/K_0.5_^F6P^) of 0.14 s^−1^·μm ^−1^ and K_0.5_^F6P^ of 178 ± 17 µm.

### Substrate affinities as measured by surface plasmon resonance

For substrate‐affinity measurements by SPR, sensor chip surfaces of 1400–3200 response units (RU) were produced for each of the three trypanosomatid PFKs. A range of substrates and other analytes were tested against each surface. Steady‐state affinities for ligands (ATP, F6P, AMP and ADP) are shown in Table 4, alongside selected combinations.

Using AMP titrations as analyte solution showed that AMP binds to TbPFK with a higher affinity (K_d_^AMP^ 0.47 mm) compared with TcPFK (1.04 mm) and LiPFK (2.3 mm). The effect of ATP on AMP binding was measured by including 1 mm ATP in both the running buffer and the AMP analyte solutions. This greatly weakened the affinity for AMP in both TbPFK (1.93 mm) and LiPFK (12.2 mm) and, to a lesser extent, TcPFK (1.69 mm). ADP binds relatively tightly to TbPFK (K_d_^ADP^ 0.56 mm) and TcPFK (0.73 mm), with LiPFK having the strongest affinity (0.16 mm). Addition of 1 mm ATP to the analyte solution decreased these affinities by three‐ to fivefold in all three enzymes, presumably due to competition in the active site (Table 4). This suggests that ADP binds mainly to the active site rather than any putative allosteric site.

Measured steady‐state affinities for ATP were strong for TcPFK, TbPFK and LiPFK (K_d_^ATP^ 35.4 µm, 15.8 and 16.8 µm, respectively) (Fig. S2). Addition of 1 mm AMP to the analyte solution did not significantly change K_d_^ATP^ (Table 4), K_d_^F6P^ (data not shown) or K_d_^ADP^ (data not shown), though tethering of the enzyme to the SPR chip surface may prevent conformational changes necessary for AMP effects. No binding response was observed for F6P, even at concentrations of up to 10 mm, against any of the trypanosomatid PFK surfaces. However, adding 1 mm ATP to the analyte solution allowed F6P binding to be observed, though the binding response did not achieve saturation even at 10 mm F6P (*R*_max_ 5.8 RU compared to the maximum binding response of 26 RU for the TbPFK surface). Lack of F6P saturation may be explained by dynamic movement of PFK while catalysing the conversion of substrates into products, or by enzyme tethering effects. The difference in F6P binding with and without ATP strongly suggests that F6P binding is contingent on ATP binding.

### *Leishmania* PFK melts at a significantly lower temperature than the *Trypanosoma* PFKs

*T. brucei*, *T. cruzi* and *L. infantum* PFKs have melting temperatures (*T*_m_) of 59 ± 0.2, 54 ± 0.2 and 39 ± 0.3 °C, respectively (see the Materials and methods and Fig. S3). The melting traces for the three trypanosomatid PFKs show that each protein melts in a single unfolding event with the rather surprising result that LiPFK melts 20 °C lower than TbPFK. The effect of a range of metabolite molecules on PFK melting temperatures was also tested (Fig. S4). The largest effect was observed with the effector AMP, which increased *T*_m_ by between 9 °C (LiPFK) and 6 °C (TbPFK). F6P does not cause a change in the *T*_m_ of any of the trypanosomatid PFKs, a result that is in line with the SPR data and supports the idea that F6P does not bind in isolation.

### The structure of AMP‐bound *T. brucei* PFK adopts a compact stable conformation

**Figure 3:**
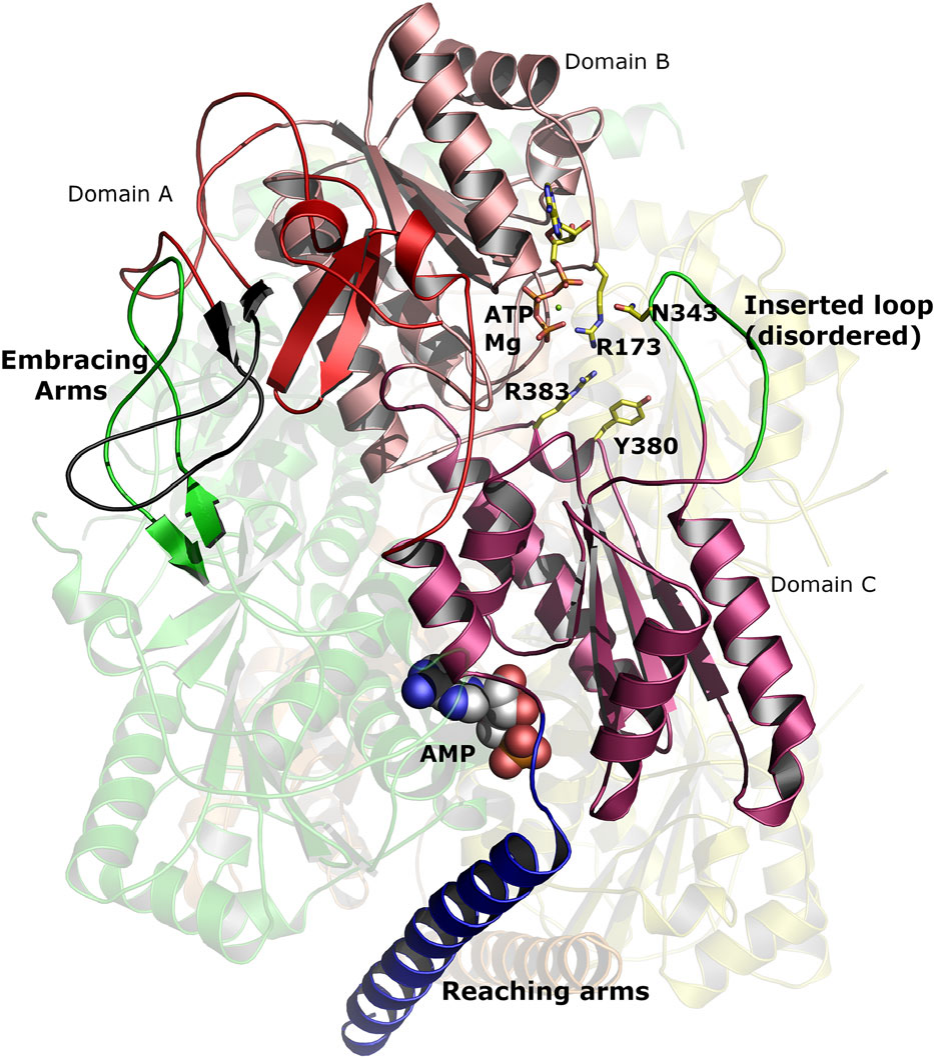
Overall structure of tetrameric *Trypanosoma brucei* PFK. Three of the four monomers are shown faded in the background and coloured yellow, orange and green. The fourth monomer is coloured in different shades of red to highlight the different domains. The ordered ‘embracing’ and ‘reaching’ arms are coloured black and blue, respectively. (The partner ‘embracing’ arm from the green monomer is also highlighted to show how the arms intertwine.) The disordered inserted loop is coloured green and taken from the ATP containing structure 3F5M 5, 24. ATP (drawn as sticks) is also taken from 3F5M. The AMP is shown as spheres with grey carbons. Graphic created with The PyMOL Molecular Graphics System (version 1.8.6.2, Schrödinger, LLC, New York, NY, USA).

The crystal structure of *T. brucei* PFK with bound AMP contains two tetramers within the asymmetric unit and has been refined at a resolution of 2.75 Å. The overall subunit architecture (Fig. 3) is very similar to that described for the apoenzyme and the ATP‐bound forms of *T. brucei* PFK (PDB 2HIG and 3F5M) 6, 19. The monomer is folded into three domains: Domain A (residues 8–94 and 410–441), Domain B (residues 95–233 and 386–409) and Domain C (residues 234–385 and 442–453) (Fig. 3).

Comparison of the AMP‐bound TbPFK structure with the apo TbPFK (PDB 2HIG) structure shows that AMP binding in the effector site results in a tightening of the tetramer and a dramatic ordering of the long helical ‘reaching arms’ (residues 458–486) which act as clamps anchoring together each pair of diametrically opposite chains in the tetramer (highlighted in Fig. 3). A similar ordering of the C‐terminal ‘reaching arm’ helices was observed in the ATP‐bound structure (PDB 2HIG), though the helices slide past each other by a full two helical turns in the AMP structure compared to the ATP structure (Supplementary Movie). The ‘embracing arms’ (residues Pro63 to Pro79) are also ordered in the AMP‐bound structure with short stretches of antiparallel β‐sheet contributing to stabilization of each dimer (Fig. 3). The ‘inserted loop’ which interacts with ATP is ordered in the ATP‐bound structure (PDB 3F5M) but remains disordered in both the AMP‐bound and apo structures (Fig. 3). A movie morphing between the apo, ATP‐bound and AMP‐bound structures shows the conformational differences between these three structures (Supplementary Movie).

**Figure 4:**
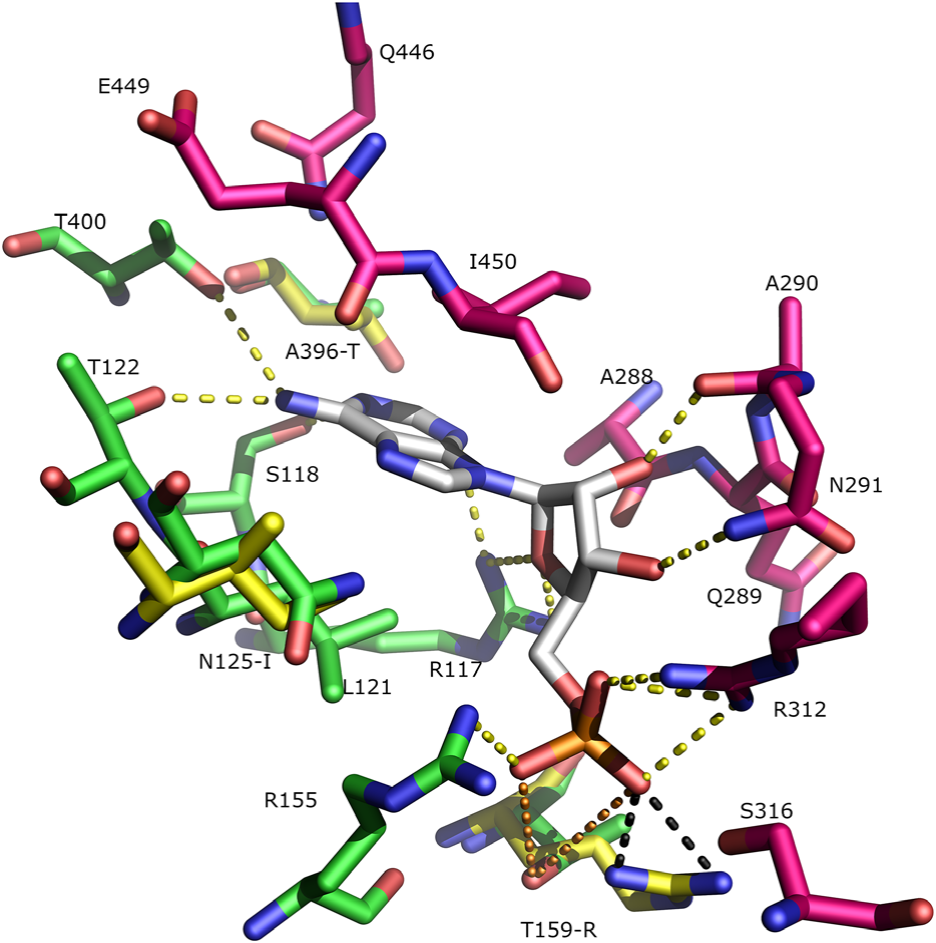
AMP binding site in *Trypanosoma brucei* PFK (TbPFK) and *Leishmania infantum* PFK (LiPFK). The X‐ray structure of the AMP binding site in TbPFK is shown with AMP coloured grey. Green and magenta residues are from different TbPFK monomers. Hydrogen bonds between AMP and TbPFK are shown as yellow dashed lines. An *L. infantum* PFK model was threaded onto the TbPFK template. Yellow residues indicate *L. infantum*‐specific changes. Black dashes show the modelled hydrogen bond interactions from (Li)Arg157 to (PO_4_)^2−^. The corresponding residue in LiPFK (Thr159) does not make favourable hydrogen bonds to AMP in the crystal (orange dashed lines > 4 Å). Graphic created with The PyMOL Molecular Graphics System (version 1.8.6.2, Schrödinger, LLC).

**Figure 5:**
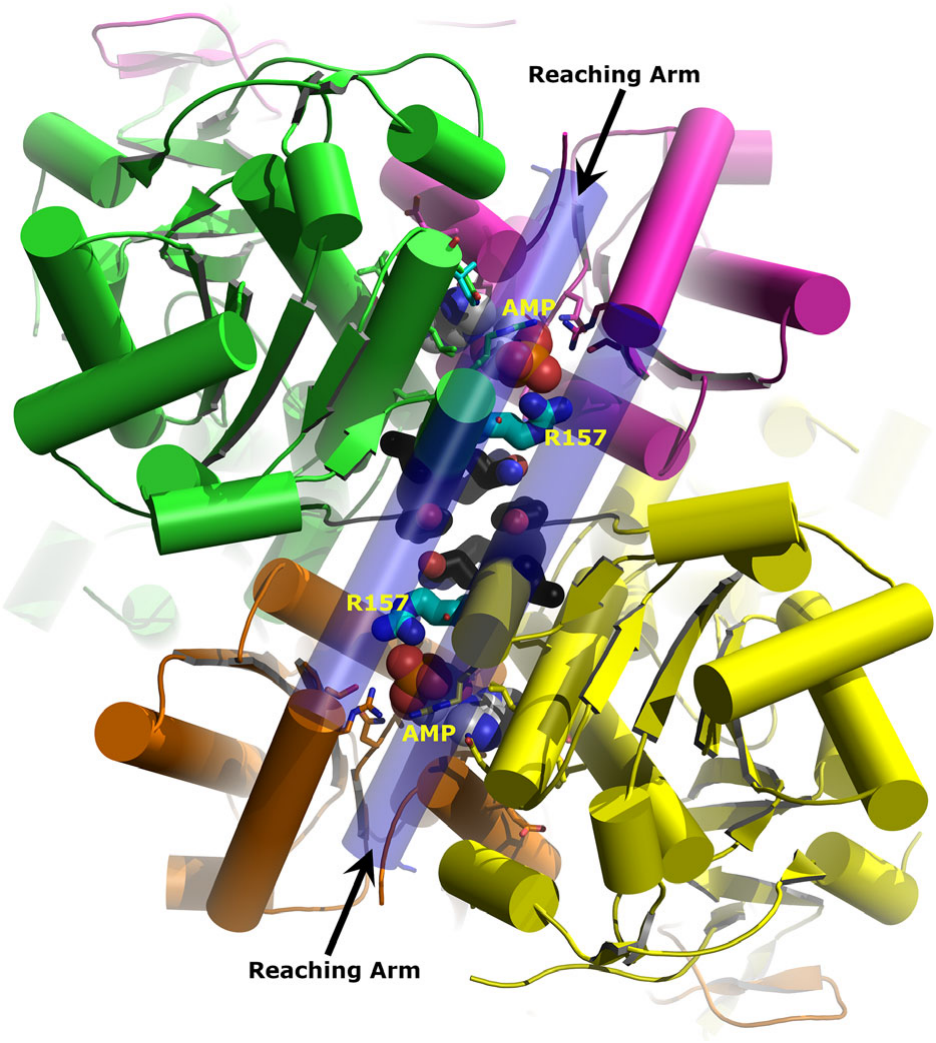
Model of LiPFK based on the X‐ray structure of *Trypanosoma brucei* PFK (TbPFK). The view is along the twofold axis relating Dimer 1 to Dimer 2. Dimer 1 is coloured green and magenta and Dimer 2 orange and yellow. AMP is shown as spheres within each monomer–monomer interface. Arg157 is shown as thick sticks with cyan carbons. Residues adjacent to Arg157 and found within the dimer–dimer interface are shown as thick sticks with black carbons. The long helical ‘reaching’ arms are shown as slightly transparent and form a lid over the AMP sites and stretch across the dimer–dimer interface. Graphic created with The PyMOL Molecular Graphics System (version 1.8.6.2, Schrödinger, LLC).

The AMP effector site is located at the interface between two monomers, approximately 30 Å from the ATP binding site. AMP forms direct interactions with pairs of monomers (Figs 3, 4, 5) resulting in closure of this interface (Figs 4 and 5). Interestingly, the binding mode of AMP in the *T. brucei* effector site, is almost 180° rotated from the pose adopted by ADP which acts as an allosteric activator in the equivalent effector site of *E. coli* PFK (PDB 1PFK) which lacks the C‐terminal extended ‘reaching arms’.

The PFK residues that form the allosteric AMP binding site are generally well conserved (11 out of 17 residues) in all Kinetoplastea analysed. There are, however, some important amino acid differences between PFKs from *Leishmania* and *Trypanosoma* that may contribute to differences in the effect of AMP on enzymatic activity that we have observed. Figure 4 shows the residues contacting AMP in TbPFK with three key substitutions that distinguish TbPFK from LiPFK (numbering of LiPFK residue shown in brackets): A396T(394), N125I(123) and T159R(157). The SPR results suggest that AMP binding to LiPFK is nearly fivefold weaker than to TbPFK.

### Comparison of PFK sequences from *Leishmania* and *Trypanosoma*

The insights from amino acid differences at the AMP binding site prompted us to see if there were more systematic differences between species belonging to *Leishmania* and *Trypanosoma*. Four diverse species from each genus were selected for comparison, including three *Leishmania* from diverse subgroups/complexes (*Leishmania*, *Sauroleishmania* and *Viannia*) and the related *Endotrypanum* (which clusters alongside *Leishmania* in phylogenetic analyses). For *Trypanosoma* species, two salivarian and two stercorarian species were selected. The amino acid sequence alignment (Fig. S1) shows 58% of amino acids are completely conserved between both groups. Of the remaining amino acids, 29% are conserved among the selected four *Leishmania* species while only 12% are conserved among the four *Trypanosoma* species (as highlighted in Fig. S1). Only 8% of the residues do not show clustering (i.e. neither the 4 *Leishmania* nor 4 *Trypanosoma* have conserved amino acids). Interestingly, the region that shows the most obvious differences between the *Leishmania* and *Trypanosoma* sequences are residues 458–486 that define the C‐terminal helical ‘reaching arm’ previously identified as playing a key role in stabilizing the tetramer and regulating the T‐to‐R allosteric transition 6, 32. Figure S1 shows that the ‘reaching arm’ helix has evolved differently in *Trypanosoma* species compared to *Leishmania* species. In the *Leishmania* group 76% of amino acids in the helix are conserved, but only 25% are conserved within the *Trypanosoma* group (with 14% being fully conserved between *Leishmania* and *Trypanosoma*).

Comparison of the *Trypanosoma* and *Leishmania* sequences (Fig. S1) also reveals differences in the ‘embracing arm’ region, comprising residues 44–75, which has previously been suggested to govern tetramer stability 6. A similar pattern of differences in conservation between the *Trypanosoma* and *Leishmania* sequences is observed: 63% of *Leishmania* group amino acids are conserved and different from *Trypanosoma* residues; only 6% are conserved in *Trypanosoma* and different from *Leishmania* residues; and 31% are conserved between both groups.

## Discussion

Previously published results on multiple trypanosomatid PFKs are summarized in Table 2. There are marked variations in reported kinetic parameters, with differences in the degree and efficiency of the purification methods and the conditions used in the activity assays. In our study, we have used similar purification protocols and assay conditions, allowing direct comparison of kinetic data from the three orthologous PFKs.

### The allosteric mechanism of AMP in trypanosomatid PFKs

**Figure 6:**
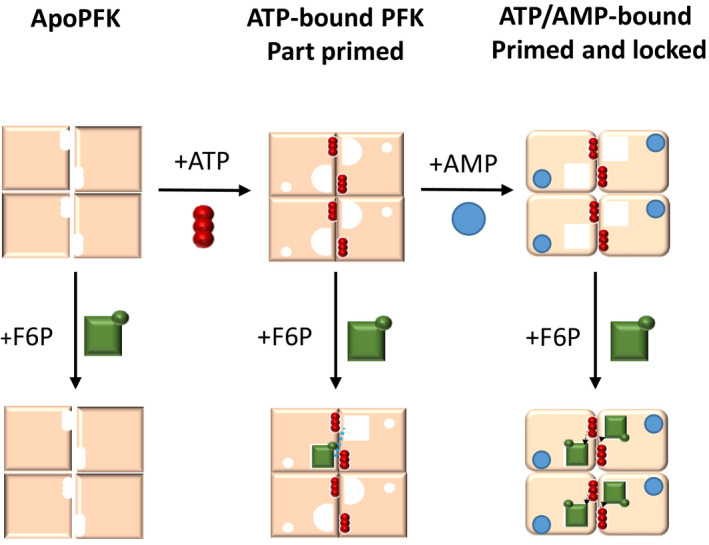
Proposed model for sequential substrate and allosteric AMP binding by trypanosomatid PFKs. ApoPFK has low affinity for fructose 6‐phosphate (F6P) (green squares) in the absence of ATP (red circles). When ATP is bound to PFK, the enzyme becomes part primed and binds F6P with medium affinity. ATP‐bound PFK permits avid binding of AMP (blue circles), allowing the enzyme to become primed and locked, with high affinity for F6P.

The finding that pre‐incubation of TbPFK with ATP before addition of F6P causes a 50% reduction in K_0.5_^F6P^ suggests that ATP binding primes the PFK tetramer and aids F6P binding. The prebinding of ATP also seems to be a requirement for the ‘allosteric activator’ effect of AMP as reduction of K_0.5_^F6P^ and an increased k_cat_/K_0.5_ is only observed when ATP is incubated with PFK before adding AMP. These observations suggest a model (Fig. 6) in which binding of ATP is key to switching the inactive T‐state conformer to an active R‐state. Our additional biophysical data for ATP binding, namely micromolar *K*_d_ measured by SPR (Table 4), a large increase in *T*_m_ (Fig. S4) and an ordering of the ‘embracing and reaching’ arms observed in the crystal structure (PDB 3F5M), all support this model. AMP binding to PFK mirrors the ATP data: *K*_d_ values (in the absence of ATP) are in the low millimolar range (Table 3); there are increases in *T*_m_ of 6–9 °C (Fig. S4) and there is ordering of the embracing and reaching arms observed in the crystal structure (this work).

The kinetic data (Table 3) suggest that AMP binding has similar characteristics to an ‘uncompetitive inhibitor’ which reduces both substrate *K*_d_ and *V*_max_ (Table 3). Also in common with uncompetitive inhibitors, AMP can only show an effect on enzyme activity once the ATP‐PFK (E‐S) complex has formed. Given the large increase in *T*_m_ upon AMP binding, we speculate that the reduction in both *K*_0.5_ and *V*_max_ caused by AMP binding is a result of the rigidification of the tetramer: ordering of the ‘reaching and embracing’ arms leading to a better‐formed substrate binding site (giving lower K_0.5_ values) and possibly slower product off rate (leading to a lower *V*_max_). Comparing the action of AMP to that of an ‘uncompetitive inhibitor’ may appear to contradict its role as an allosteric activator; however, the parasite PFKs will be operating under physiological conditions and AMP will indeed enhance rate when substrate concentrations are around the measured K_0.5_ values. The reduction of *V*_max_ in the presence of AMP will only become relevant at much higher, nonphysiological values of substrate.

### How can the enhanced allosteric action of AMP in LiPFK be explained?

In the light of the sequence analysis showing that most of the sequence differences between the *Leishmania* and *Trypanosoma* species are in the stabilizing ‘reaching arm’ and ‘embracing arm’ regions, it is reasonable to suggest that these differences explain the striking (> 15 °C) lowering of melting temperature of LiPFK compared with the two *Trypanosoma* PFKs. The three available structures (apo, ATP‐bound and AMP‐bound) also show that addition of ATP or AMP has a dramatic effect on ordering these two (‘reaching and embracing’) regions. The rise in melting temperature on addition of these ligands (6–9 °C for AMP and 4–6 °C for ATP) is also consistent with the observed increase in secondary structure and enhanced interchain interactions in the tetramer. The kinetic data for trypanosomatid PFKs fit with the standard allosteric model for the tetramer which transitions from an inactive ‘T‐state’ conformation (apo structure PDB 2HIG with disordered stabilizing regions) to an active R‐state conformation (more similar to the AMP or ATP‐bound structures). Compared with the *Trypanosoma* PFKs, the low‐melting LiPFK protein will favour the partially disordered and inactive T‐state which requires the stabilizing effect of AMP (disproportionally more than the higher melting *Trypanosoma* PFKs) before it can adopt the R‐state conformation.

### The regulatory roles of trypanosomatid PFKs in glycolysis

Metabolic control analysis studies with bloodstream‐form *T. brucei* show that PFK has no detectable flux control; its activity is in excess compared to most of the other enzymes (except of hexokinase) 33. If this is also the case for other trypanosomatid species, it could be argued that the *Trypanosomas* have evolved to remove all flux control from PFK, irrespective of the F6P concentration in the cell. In contrast, *Leishmania* PFK (which is up to 15‐fold less efficient than the *Trypanosoma* PFKs) may control flux at low F6P concentrations, but this control may be released under certain conditions by increasing the glycosomal AMP concentration. Such a regulatory mechanism may be linked with the ability of *Leishmania* spp. amastigotes residing in the phagosomes of macrophages to enter a metabolically quiescent state, thought to minimize immune activation 34. Intracellular *Leishmania* amastigotes rely on glycolysis for their ATP production 27. The ability to downregulate the activity of a glycolytic enzyme like PFK to switch from replicating to dormant amastigotes and *vice versa*, dependent on conditions and/or escape into the macrophage’s cytosol, would thus makes sense. Direct measurements of adenine nucleotide concentrations in glycosomes are technically difficult, but models predict [AMP] < [ADP] < [ATP] 30; thus, AMP concentrations are a more sensitive indicator of low‐energy states than other nucleotide concentrations. Another consequence of the strong F6P‐dependent activity regulation by AMP may be that at low glycosomal AMP concentration, a major proportion of hexoses consumed may be rerouted away from glycolysis to the pentose‐phosphate pathway.

### The evolution of Trypanosomatidae PFKs

A genome‐wide analysis of evolution of the Trypanosomatidae family suggests a split between the lineages leading to the *Trypanosoma* and *Leishmania* genera occurred about 120 million years ago 22, 23. Our sequence analysis (Fig. S1) shows that residues forming both ‘reaching arms’ and ‘embracing arms’ (responsible for stabilizing the PFK tetramer) are much better conserved between the four *Leishmania* species than between the equivalent stabilizing regions of the four *Trypanosoma* species. These sequence differences correlate with the lower stability (lower *T*_m_) measured for *Leishmania* PFK and provide a rationale for the different allosteric effect of AMP on enzyme kinetics of *Leishmania* and *Trypanosoma* PFKs. The greater divergence from the common ancestral PFK sequence by *Trypanosoma* species strongly suggests that AMP regulation was present in the early common ancestor of the two genera but that the *Trypanosoma* branch has a lesser need for this regulatory mechanism while during the course of evolution *Leishmania* PFKs have retained AMP regulation.

### Are differences in AMP‐regulated activity between *Leishmania* and *Trypanosoma* consistent with parasite environment?

*Leishmania* lives intracellularly in macrophages and is therefore only exposed to low glucose levels (only trypomastigote forms being present extracellularly in vertebrate hosts during short periods may encounter high glucose concentrations). In its insect vector, the sandfly, it will only occasionally have access to sugars. Nonetheless, expression of glycosomal glycolytic enzymes is not downregulated; their protein levels are even slightly increased 35 and our data suggest that AMP may play an important role in regulating this pathway. At high AMP concentrations, LiPFK will have high activity irrespective of the F6P concentration; however, at low AMP concentrations, LiPFK will be much less active.

Although *T. cruzi* amastigotes are also known to be capable of entering into a dormant stage during a chronic phase of Chagas disease 36, they seem to follow a different strategy than *Leishmania*. Intracellular *T. cruzi* reside in the host cell’s cytosol where the concentration of glucose and other sugars is very low. They do not rely on glucose 26 and differ from *Leishmania* amastigotes in that expression of their glycolytic enzymes is repressed 37, suggesting less need for activity regulation of the enzymes, *T. brucei* differs from *T. cruzi* and *Leishmania* spp. by living extracellularly throughout its life cycle, during which it encounters abundant glucose in the mammalian bloodstream, but hardly any in the intestinal tract of the tsetse fly, except for very short periods after the insect has taken a bloodmeal. This is reflected in high expression of glycolytic enzymes in the bloodstream form, whereas expression of many of the enzymes is considerably downregulated in the procyclic insect form 38. Nonetheless, glucose appears to be preferred as substrate over amino acids by procyclic forms when glucose is available, even at low concentration 39. Glycolytic flux in bloodstream and procyclic forms does not appear to be regulated by allosteric activators or inhibitors for any of the enzymatic steps 33, 40. These observations on *T. brucei* and those mentioned above for *T. cruzi* are consistent with our finding that in *Trypanosoma* activity regulation of PFK by AMP is minimal; glycolysis is active at a wide range of F6P concentrations, irrespective of AMP concentration and gluconeogenesis will therefore only be favoured at very low F6P concentrations. Thus, *Trypanosoma* still prefers the use of glucose for ATP production, even when the glucose supply is low and gluconeogenic substrates (amino acids, glycerol) that can also be used for ATP production are abundantly present, notably in its insect vectors.

### AMP acts as a switch between glycolysis and gluconeogenesis for *Leishmania*

Interestingly, the gluconeogenic enzyme fructose‐1,6‐bisphosphatase (FBPase) (EC3.1.3.11) is regulated differently in the different kinetoplastid families. This enzyme, which like PFK is in glycosomes, carries out the reverse reaction to PFK (converting F16BP to F6P + Pi). It is present in both life‐cycle stages of *T. brucei*. No FBPase activity is found in lysates of glucose‐grown bloodstream‐form trypanosomes, strongly suggesting that it is regulated by post‐translational modification (PM, unpublished data). Previously, we reported that leishmanial FBPase is strongly regulated by AMP – but in the opposite direction to PFK – with rising concentrations of AMP inhibiting gluconeogenesis (IC_50_ 63.8 µm for *Leishmania major* FBPase) 41. Our data presented here on the activation of LiPFK by AMP, combined with the previously published data on AMP inhibition of LmFBPase, strongly imply that in *Leishmania*, AMP plays a role in controlling the balance between glycolysis and gluconeogenesis with AMP concentration providing a switching mechanism that can simultaneously shut down gluconeogenesis and upregulate glycolysis. As we have shown here, AMP is a much less efficient activator of *Trypanosoma* than *Leishmania* PFK. It is reasonable to suggest that sensitivity of the AMP‐regulated switch to adjust the glycolytic flux to glucose availability in *Leishmania*, as well as to govern the balance between glycolysis and gluconeogenesis, is an important factor in the evolutionary divergence of *Leishmania* and *Trypanosoma* that is consistent with their different biological niches.

## Materials and methods

Recombinant trypanosomatid PFKs were expressed in *E. coli* and purified using the method described previously 42; full sequence alignments are shown in Fig. S1.

### Determination of kinetic characteristics using pyruvate kinase–lactate dehydrogenase enzyme‐linked kinetic assay

Kinetic values for PFK were determined using an *in vitro* assay connecting ADP production to NADH oxidation (measurable with UV absorbance at A_340nm_) through pyruvate kinase and then lactate dehydrogenase.

Assay buffer consisted of 50 mm TEA, 100 mm KCl, 10 mm MgCl_2_ and 10% glycerol, pH 7.4. Assay mix consisted of 1.2 mm NADH (Sigma‐Aldrich N4505, Gillingham, Dorset, UK), 33 units·mL^−1^ lactate dehydrogenase (Sigma‐Aldrich L1254), 4.8 mm phosphoenolpyruvate (P0564) and 20 units·mL^−1^ human M1 pyruvate kinase in assay buffer (expressed and purified from *E. coli* in the Edinburgh Protein Production Facility, University of Edinburgh). ATP and F6P were obtained from Sigma‐Aldrich (A2383 and F3627, respectively). Trypanosomatid PFKs were added to the assay mix, at final concentrations of 4 μg·mL^−1^ for TcPFK and TbPFK and 8.7 μg·mL^−1^ for LiPFK (higher enzyme concentrations were required for LiPFK experiments due to lower intrinsic activity). All ligands and substrates (including ATP) were used at pH 7.4. All metabolites were obtained from Sigma‐Aldrich, including AMP (O1930) and GTP (G8877).

Forty microlitre assay mix with PFK was added to a clear nonbinding 96‐well plate. Twenty microlitre of ATP was added, to give a final concentration of 1.5 mm. Alternatively, 20 μL of ATP titration was added, to give final concentrations of 2.5 mm downwards (8–12 serial 50% dilutions). Twenty microlitre of ligand (final concentration stated in text) or buffer was added, and the plate was incubated at 25 °C for 5 min. F6P was added last, to start the reaction, with 20 μL of F6P (final concentration 3 mm) or F6P titration, to give final concentrations of 5 mm downwards (8–12 serial 50% dilutions). Reaction order was kept constant unless otherwise stated. Concentration of NADH, assessed via A_340nm,_ was measured at 12‐s intervals for 10 min using a SpectraMax M5 Multi‐Mode Microplate Reader.

The Beer–Lambert law was used to convert time‐dependent absorbance change into rate of NADH oxidation (μm·s^−1^) or specific activity (μmol·min^−1^·mg protein^−1^), using NADH’s molar extinction coefficient of 6.22 mm ^−1^·cm^−1^. Reaction rates were calculated using a 4‐point (48 s) rolling average. GraphPad Prism 7 was used for data analysis. Kinetic properties were determined using nonlinear regression analysis of substrate titration data to generate curves fitted using allosteric sigmoidal model calculated using the following equation:

$$V_{0}=V_{\max}\left(\frac{X^{h}}{X^{h}+K_{m}^{h}}\right).$$

### Surface plasmon resonance (SPR) assays for measuring analyte binding to trypanosomatid PFKs

Sensor chip surfaces with active N‐terminal His_6_‐tagged *T. brucei*, *T. cruzi* and *L. infantum* PFK were generated by using a capture/stabilization method 43, 44. Surface densities corresponding to 1400–3200 resonance units (RU) were immobilized on Ni^2+^‐nitrilotriacetic acid sensor chips (GE Healthcare, GE Healthcare Life Sciences, Little Chalfont, Buckinghamshire, UK). Substrates and other metabolic analytes were made up and buffered in HBS buffer (10 mm HEPES, 150 mm NaCl, 10 mm MgCl_2_, 0.05% Tween‐20, pH 7.4). The following analytes were made up in 2.5‐fold serial dilutions over concentration ranges 10 mm to 7.8 µm: AMP, ADP, ATP, GTP, GDP, F6P, F16BP; concentration ranges 20 mm to 156 µm were used for citrate, inorganic pyrophosphate, phosphoenolpyruvate. Analytes were washed over the surfaces at a rate of 30 µL·min^−1^ with 30‐second contact time and washed off with HBS buffer for 30 s. When analytes were tested in the presence of AMP or ATP, these analytes were added to a concentration of 1 mm to the HBS running buffer and all analyte dilution series were made up in this buffer. All values were calculated from plots of steady‐state responses versus concentration using analysis software (v2.02) provided with the BIAcore T200 instrument (GE Healthcare).

### Thermal denaturation measurements of trypanosomatid PFK

Thermal denaturation assays (TDA) were carried out to assess the thermal stability of the trypanosomatid PFKs. A real‐time PCR instrument was used (Bio‐Rad iQ™5 iCycler, Bio‐Rad Laboratories, Ltd. Watford, UK) to heat the PFK samples from 20 to 80 °C in the presence of a fluorescent dye – ‘SYPRO Orange’ (Sigma). Upon denaturation of the protein, the SYPRO Orange dye binds the exposed hydrophobic regions, and the increase in fluorescent signal (ex/em = 485/575 nm) is measured in relative fluorescence units (RFU).

The transition melting temperatures (*T*_m_ values) of *T. brucei*, *T. cruzi* and *L. infantum* PFKs were determined using the same TEA‐based buffer (10 mm MgCl_2_, 100 mm KCl, 50 mm TEA, 10% glycerol, 0.005% Tween, pH 7.4). 50‐µL reactions containing 1.9 µm PFK were heated with 0.5 °C increments from 4 to 80 °C in the presence of SYPRO Orange.

### Crystallization and structure determination and model building

Purified *T. brucei* PFK in gel filtration buffer containing 1 mm F16BP, 1 mm AMP and 500 µm novel PFK inhibitor 32 was concentrated to 6 mg·mL^−1^ and crystallized via hanging drop methods at 18 °C. The well solution consisted of 9.5% PEG 8000 and 0.1 m sodium cacodylate at pH 7.4. Drops were set up containing 1 µL protein solution, 1.5 µL well solution and 0.5 µL silver bullet 23 (Hampton Research, 0.25% w/v 1,2‐diaminocyclohexane sulfate, 0.25% w/v 1,4‐cyclohexanedicarboxylic acid, 0.25% w/v methylenediphosphonic acid, 0.25% w/v sulfanilic acid and 0.02 m HEPES sodium, pH 6.8). Data were collected on beamline I04‐1 at the Diamond synchrotron radiation facility. The intensity data were collected from single crystals flash‐frozen in liquid nitrogen at 100 K. Data were processed with XDS and AIMLESS 45. Initial phases were obtained by molecular replacement using the program PHASER 46 and 3F5M as the search model. Refinement was performed with the program REFMAC utilizing local NCS restraints. Manual adjustment was performed using the program COOT 47 as part of the CCP4 program suite 48, and data collection and refinement statistics are shown in Table S1. Noncrystallographic restraints were not applied during refinement and each of the eight PFK chains in the asymmetric unit was refined independently. Although crystallization was performed in the presence of AMP, F16BP and novel PFK inhibitor, only density for AMP could be identified and modelled. A small amount of unidentified density was found near the ATP binding site and has been modelled with a benzene ring. A lower resolution (3.5 Å) structure (data not shown) with only AMP bound in the effector site has an RMSD of less than 0.6 Å to the structure presented here; thus, we can be confident that this structure and its allosteric movement are a reflection solely of the binding of AMP to TbPFK without significant influence of the inhibitor or F16BP. A model for LiPFK was produced using the Phyre2 Web portal 49 and its one‐to‐one threading mode. The AMP structure described here was used as the input model.

## Conflicts of interest

The authors declare no conflict of interest.

## Author contributions

PMF, JK, IWM, MAW, PAMM and MDW were involved in the conception and design of the experiments and interpretation of the data. MV‐V contributed reagents. PMF and JK performed the experiments and prepared the first draft of the manuscript. PMF, JK, PAM and MDW wrote the final version of the manuscript.

## Supporting information


**Fig. S1.** Amino acid sequence comparisons for phosphofructokinase from four trypanosomal and four leishmanial‐related species.
**Fig. S2.** Surface Plasmon Resonance sensorgrams for TbPFK, TcPFK, and LiPFK.
**Fig. S3.** Thermal denaturation assays of trypanosomatid PFKs.
**Fig. S4.** Melting temperature shifts for trypanosomatid PFKs with substrates.
**Table S1.** Crystallographic data for *Trypanosoma brucei* phosphofructokinase (PDB code 6SY7. Values in parentheses are for the highest resolution shell.
