# Supplementary material for: Kinetic and structural studies of Trypanosoma and Leishmania phosphofructokinases show evolutionary divergence and identify AMP as a switch regulating glycolysis versus gluconeogenesis
Source: FEBS J. 2020 Jan 8;287(13):2847–61. doi: 10.1111/febs.15177 (PMC7383607; doi:10.1111/febs.15177)
Supplement: Supplementary file 1 — Fig. S1. Amino acid sequence comparisons for phosphofructokinase from four trypanosomal and four leishmanial‐related species. Fig. S2. Surface Plasmon Resonance sensorgrams for TbPFK, TcPFK, and LiPFK. Fig. S3. Thermal denaturation assays of trypanosomatid PFKs. Fig. S4. Melting temperature shifts for trypanosomatid PFKs with substrates. Table S1. Crystallographic data for Trypanosoma brucei phosphofructokinase (PDB code 6SY7. Values in parentheses are for the highest resolution shell. [file FEBS-287-2847-s001.zip › febs15177-sup-0001-Supinfo.pdf]

FEBS PRESS
science publishing by scientists
WILEY
The FEBS Journal
www.febsjournal.org

Kinetic and structural studies of *Trypanosoma* and *Leishmania* phosphofructokinases show evolutionary divergence and identify AMP as a switch regulating glycolysis *versus* gluconeogenesis

Peter M. Fernandes, James Kinkead, Iain W. McNae, Monserrat Vásquez-Valdivieso, Martin A. Wear, Paul A. M. Michels and Malcolm D. Walkinshaw

DOI: 10.1111/febs.15177

Figure Supplementary 1 (Figure S1)

Amino acid sequence comparisons for phosphofructokinase from four trypanosomal and four leishmanial-related species. Blue indicates shared identity between PFKs of all eight species. Green indicates shared identity between trypanosomal species only. Red indicates shared identity between leishmanial-related species only. Bold are ATP binding site residues, italic are F6P binding site residues (McNae et al., J. Mol. Biol. 2009, 385, 1519–1533). Amino acid sequences were retrieved from www.uniprot.org; alignments made using Clustal Omega 2.1 (http://www.ebi.ac.uk/Tools/msa/clustalo/). Accession numbers: *Trypanosoma brucei* PFK (O15648); *Trypanosoma congolense* PFK (F9W8G2); *Trypanosoma cruzi* PFK (Q4E657); *Trypanosoma rangeli* PFK (A0A3R7N9H0); *Leishmania infantum* PFK (A4I4W5); *Leishmania tarentolae* PFK (LtaP29.2710); *Leishmani braziliensis* PFK (A4HHQ0); *Endotrypanum monterogeii* PFK (EMOLV88_290005100).

Species	-2	-1	1	2	3	4	5	6	7	8	9	10	11	12	13	14	15	16	17	18	19	20	21	22	23	24	25
Trypanosoma brucei	M	A	V	E	S	R	S	R	V	T	S	K	L	V	K	A	H	R	A	M	L	N	S	V	T	Q	E
Trypanosoma congolense	M	A	L	D	S	R	S	R	I	T	S	K	L	V	K	A	H	Q	A	M	L	H	S	V	T	Q	D
Trypanosoma cruzi	-	-	M	E	N	R	L	R	D	T	S	R	V	V	R	S	H	A	A	P	L	N	E	V	T	Q	E
Trypanosoma rangeli	-	M	T	E	N	R	L	R	V	T	S	T	L	V	R	S	H	Q	A	P	L	S	V	V	T	Q	E
Leishmania infantum	-	-	M	E	T	R	H	H	L	N	T	K	M	V	P	S	Y	Q	A	P	L	S	K	V	T	A	A
Leishmania tarentolae	-	-	M	E	T	R	R	Y	L	N	T	K	M	V	P	S	H	Q	A	P	L	S	E	V	T	A	A
Leishmania braziliensis	-	-	M	E	T	R	S	H	I	N	T	K	M	V	P	S	Y	H	P	P	L	S	K	V	T	A	A
Endotrypanum monterogeii	-	-	M	D	T	R	Q	R	V	N	S	K	M	V	P	S	F	E	A	P	L	S	Q	V	T	A	A

Species	26	27	28	29	30	31	32	33	34	35	36	37	38	39	40	41	42	43	44	45	46	47	48	49	50	51	52
Trypanosoma brucei	D	L	K	V	D	R	L	P	G	A	D	Y	P	N	P	S	K	K	Y	S	S	R	T	E	F	R	D
Trypanosoma congolense	D	L	K	V	E	R	L	P	G	T	E	Y	P	N	P	S	K	K	Y	V	A	R	E	E	F	S	E
Trypanosoma cruzi	D	L	K	V	E	R	L	H	G	R	K	Y	M	N	P	S	K	K	H	V	M	R	E	E	F	S	D
Trypanosoma rangeli	D	L	K	V	L	R	L	R	G	R	K	Y	T	N	P	S	K	K	H	V	M	R	A	E	F	S	D
Leishmania infantum	D	L	T	V	E	R	L	P	G	C	K	Y	M	N	P	S	K	K	H	I	L	R	E	E	Y	R	D
Leishmania tarentolae	D	L	A	V	E	R	L	P	G	C	K	Y	M	N	P	S	K	K	H	I	L	R	E	E	Y	R	D
Leishmania braziliensis	D	L	T	V	E	R	L	P	G	C	K	Y	M	N	P	S	K	K	H	I	L	R	E	E	Y	R	D
Endotrypanum monterogeii	D	L	T	V	E	R	L	P	G	A	K	Y	E	N	P	S	K	K	H	I	L	R	E	E	Y	R	D

Species	53	54	55	56	57	58	59	60	61	62	63	64	65	66	67	68	69	70	71	72	73	74	75	76	77	78	79
Trypanosoma brucei	K	T	D	Y	I	M	Y	N	P	R	P	R	D	E	P	S	S	E	N	P	V	S	V	S	P	L	L
Trypanosoma congolense	K	I	D	Y	I	M	Y	N	P	R	P	K	D	E	V	S	S	G	N	P	V	S	A	S	P	L	L
Trypanosoma cruzi	K	I	E	H	I	M	H	D	P	R	P	Q	E	G	V	H	S	E	L	P	V	S	I	S	P	L	L
Trypanosoma rangeli	K	I	D	H	I	M	Y	D	P	R	P	K	E	G	A	D	S	E	V	P	I	S	M	S	P	L	L
Leishmania infantum	K	V	E	H	I	M	Y	D	P	R	P	Q	E	D	L	D	A	E	Y	P	V	S	C	N	K	L	V
Leishmania tarentolae	K	V	E	H	I	M	Y	D	P	R	P	Q	E	D	L	D	A	E	Y	P	V	S	C	N	K	L	V
Leishmania braziliensis	K	V	E	H	I	M	Y	D	P	R	P	Q	E	D	L	D	S	E	Y	P	V	S	C	N	K	L	V

Endotrypanum monterogeii	K	V	E	H	I	M	Y	D	P	R	P	Q	E	D	L	T	S	E	Y	P	V	S	C	N	K	L	V

Species	80	81	82	83	84	85	86	87	88	89	90	91	92	93	94	95	96	97	98	99	100	101	102	103	104	105	106
Trypanosoma brucei	C	E	L	A	A	A	R	S	R	I	H	F	N	P	T	E	T	T	I	G	I	V	T	C	G	**G**	I
Trypanosoma congolense	C	E	I	A	A	A	R	S	H	I	H	F	N	P	A	E	T	T	V	G	I	V	T	C	G	**G**	I
Trypanosoma cruzi	C	E	L	A	A	P	R	Q	R	I	H	F	N	P	P	E	T	V	V	G	I	V	T	C	G	**G**	I
Trypanosoma rangeli	C	E	L	A	A	P	R	R	R	I	H	F	N	P	S	E	T	V	V	G	I	V	T	C	G	**G**	I
Leishmania infantum	C	E	L	A	A	A	R	K	H	L	H	F	N	P	S	E	T	S	I	G	I	V	T	C	G	**G**	I
Leishmania tarentolae	C	E	L	A	A	A	R	K	H	L	H	F	N	P	S	D	T	S	I	G	I	V	T	C	G	**G**	I
Leishmania braziliensis	C	E	L	A	A	A	R	K	H	L	H	F	N	P	S	E	T	S	I	G	I	V	T	C	G	**G**	I
Endotrypanum monterogeii	C	E	L	A	S	A	R	K	H	L	H	F	N	P	A	E	T	S	I	G	I	V	T	C	G	**G**	I

Species	107	108	109	110	111	112	113	114	115	116	117	118	119	120	121	122	123	124	125	126	127	128	129	130	131	132	133
Trypanosoma brucei	C	P	G	L	N	D	V	I	R	S	I	T	L	T	G	I	N	V	Y	N	V	K	R	V	I	G	F
Trypanosoma congolense	C	P	G	L	N	D	V	I	R	S	I	T	L	T	S	I	N	V	Y	N	V	K	R	V	I	G	F
Trypanosoma cruzi	C	P	G	L	N	D	V	I	R	S	L	T	L	T	A	V	N	A	Y	R	V	K	R	V	I	G	F
Trypanosoma rangeli	C	P	G	L	N	D	V	I	R	S	L	T	L	T	A	V	N	A	Y	R	V	K	R	V	I	G	F
Leishmania infantum	C	P	G	L	N	D	V	I	R	S	I	T	L	T	G	I	I	S	Y	R	V	K	R	V	V	G	F
Leishmania tarentolae	C	P	G	L	N	D	V	I	R	S	I	T	L	S	G	I	I	A	Y	R	V	K	R	V	V	G	F
Leishmania braziliensis	C	P	G	L	N	D	V	I	R	S	I	T	L	A	G	I	I	A	Y	R	V	K	R	V	V	G	F
Endotrypanum monterogeii	C	P	G	L	N	D	V	I	R	S	I	T	L	T	G	I	L	T	Y	R	V	K	R	V	V	G	F

Species	134	135	136	137	138	139	140	141	142	143	144	145	146	147	148	149	150	151	152	153	154	155	156	157	158	159	160
Trypanosoma brucei	R	F	G	Y	W	G	L	S	K	K	G	S	Q	T	A	I	E	L	H	R	G	R	V	T	N	I	H
Trypanosoma congolense	R	F	G	Y	W	G	L	S	K	K	G	S	H	T	A	I	E	L	H	R	G	S	V	T	N	I	H
Trypanosoma cruzi	R	F	G	Y	W	G	L	S	K	K	G	S	H	T	A	M	E	L	Y	R	T	S	V	T	S	I	H
Trypanosoma rangeli	R	F	G	Y	W	G	L	S	K	E	G	A	H	T	A	M	E	L	Y	R	T	S	V	T	S	I	H
Leishmania infantum	R	Y	G	Y	W	G	L	S	K	E	G	S	K	T	A	I	E	L	S	R	S	D	V	R	Q	I	H
Leishmania tarentolae	R	Y	G	Y	W	G	L	S	E	E	G	S	K	T	A	I	E	L	S	R	T	D	V	R	Q	I	H
Leishmania braziliensis	R	Y	G	Y	W	G	L	S	K	E	G	S	K	T	A	I	E	L	S	R	T	D	V	R	Q	I	H
Endotrypanum monterogeii	R	Y	G	Y	W	G	L	S	K	E	G	S	K	T	A	V	E	L	T	R	V	D	V	R	Q	I	H

Species	161	162	163	164	165	166	167	168	169	170	171	172	173	174	175	176	177	178	179	180	181	182	183	184	185	186	187
Trypanosoma brucei	H	Y	G	G	T	I	L	G	S	S	***R***	G	**P**	Q	D	P	K	E	M	V	D	T	L	E	R	L	G
Trypanosoma congolense	H	Y	G	G	T	I	L	G	S	S	***R***	G	**P**	Q	D	P	K	E	M	V	D	T	L	E	R	L	G
Trypanosoma cruzi	R	Y	G	G	T	I	L	G	S	S	***R***	G	**P**	Q	D	T	S	E	M	V	D	T	L	E	R	L	G
Trypanosoma rangeli	R	Y	G	G	T	I	L	G	S	S	***R***	G	**P**	Q	D	P	K	D	M	V	D	T	L	E	R	L	G
Leishmania infantum	R	F	G	G	T	I	L	G	S	S	***R***	G	**P**	Q	N	P	K	E	M	V	D	T	L	V	R	M	K
Leishmania tarentolae	R	F	G	G	T	I	L	G	S	S	***R***	G	**P**	Q	N	P	K	E	M	V	D	T	L	V	R	M	K
Leishmania braziliensis	R	F	G	G	T	I	L	G	S	S	***R***	G	**P**	Q	S	S	E	E	M	V	D	T	L	V	R	M	K
Endotrypanum monterogeii	R	F	G	G	T	I	L	G	S	S	***R***	G	**P**	Q	S	S	E	E	M	V	D	T	L	V	R	M	K

Species	188	189	190	191	192	193	194	195	196	197	198	199	200	201	202	203	204	205	206	207	208	209	210	211	212	213	214
Trypanosoma brucei	V	N	I	L	F	T	V	G	**G**	D	**G**	**T**	Q	R	G	A	L	V	I	S	Q	E	A	K	R	R	G
Trypanosoma congolense	V	N	I	L	F	T	V	G	**G**	D	**G**	**T**	Q	R	G	A	L	V	L	A	N	E	A	K	R	R	G
Trypanosoma cruzi	V	N	I	L	F	T	V	G	**G**	D	**G**	**T**	Q	R	G	A	L	K	I	A	E	E	A	K	R	R	G
Trypanosoma rangeli	V	N	I	L	F	T	V	G	**G**	D	**G**	**T**	Q	R	G	A	L	T	I	A	E	E	A	K	R	R	G
Leishmania infantum	I	N	I	L	F	T	V	G	**G**	D	**G**	**T**	Q	R	G	A	L	T	I	Y	E	E	A	K	R	R	G
Leishmania tarentolae	I	N	I	L	F	T	V	G	**G**	D	**G**	**T**	Q	R	G	A	L	K	I	Y	E	E	A	K	R	R	G
Leishmania braziliensis	I	N	I	L	F	T	V	G	**G**	D	**G**	**T**	Q	R	G	A	L	K	I	Y	E	E	A	K	R	R	G
Endotrypanum monterogeii	I	N	I	L	F	T	V	G	**G**	D	**G**	**T**	Q	R	G	A	L	K	L	Y	E	E	A	R	R	R	G

Species	215	216	217	218	219	220	221	222	223	224	225	226	227	228	229	230	231	232	233	234	235	236	237	238	239	240	241
Trypanosoma brucei	V	D	I	S	V	F	G	V	P	**K**	T	I	D	N	D	L	S	F	S	H	R	T	F	G	F	Q	T
Trypanosoma congolense	A	D	I	S	V	F	G	V	P	**K**	T	I	D	N	D	L	S	F	S	H	R	T	F	G	F	Q	T
Trypanosoma cruzi	A	N	L	A	V	F	G	I	P	**K**	T	I	D	N	D	L	S	F	S	H	R	T	F	G	F	E	T
Trypanosoma rangeli	T	N	I	A	V	F	G	I	P	**K**	T	I	D	N	D	L	S	F	S	H	R	T	F	G	F	E	T
Leishmania infantum	E	N	I	A	V	F	G	V	P	**K**	T	I	D	N	D	L	A	F	S	H	R	T	F	G	F	Q	T
Leishmania tarentolae	E	N	I	A	V	L	G	V	P	**K**	T	I	D	N	D	L	A	F	S	H	R	T	F	G	F	Q	T
Leishmania braziliensis	E	N	I	S	V	F	G	V	P	**K**	T	I	D	N	D	L	A	F	S	H	R	T	F	G	F	Q	T
Endotrypanum monterogeii	E	D	I	A	V	F	G	V	P	**K**	T	I	D	N	D	L	A	F	S	H	R	T	F	G	F	Q	T

Species	242	243	244	245	246	247	248	249	250	251	252	253	254	255	256	257	258	259	260	261	262	263	264	265	266	267	268
Trypanosoma brucei	A	V	E	K	A	V	Q	A	I	R	A	A	Y	A	E	A	V	S	A	N	Y	G	V	G	V	V	K
Trypanosoma congolense	A	V	E	K	A	V	Q	A	I	R	A	A	Y	A	E	A	I	S	L	N	Y	G	V	G	I	V	K
Trypanosoma cruzi	A	V	D	K	A	V	E	A	V	R	A	A	Y	A	E	A	I	S	L	N	Y	G	V	G	V	V	K
Trypanosoma rangeli	A	V	D	K	A	V	E	A	V	R	A	A	Y	A	E	A	I	S	L	N	Y	G	I	G	I	V	K
Leishmania infantum	A	V	E	Q	A	V	N	A	V	R	A	A	Y	A	E	A	V	S	L	N	Y	G	V	G	I	V	K
Leishmania tarentolae	A	V	E	Q	A	V	N	A	V	R	A	A	Y	A	E	A	V	S	L	R	Y	G	V	G	I	V	K
Leishmania braziliensis	A	V	E	Q	A	T	N	A	V	R	A	A	Y	A	E	A	V	S	L	N	Y	G	V	G	I	V	K
Endotrypanum monterogeii	A	V	E	Q	A	V	N	A	V	R	A	A	Y	A	E	S	V	S	L	N	Y	G	V	G	I	V	K

Species	269	270	271	272	273	274	275	276	277	278	279	280	281	282	283	284	285	286	287	288	289	290	291	292	293	294	295
Trypanosoma brucei	L	M	G	R	D	S	G	F	I	A	A	Q	A	A	V	A	S	A	Q	A	N	I	C	L	V	P	E
Trypanosoma congolense	L	M	G	R	D	S	G	F	I	A	A	Q	A	A	V	A	S	A	Q	A	N	I	C	L	V	P	E
Trypanosoma cruzi	L	M	G	R	D	S	G	F	I	A	A	E	A	A	V	A	S	A	Q	A	N	I	C	L	V	P	E
Trypanosoma rangeli	L	M	G	R	D	S	G	F	I	A	A	E	A	A	V	A	S	A	Q	A	N	I	C	L	V	P	E
Leishmania infantum	L	M	G	R	E	S	G	F	I	A	A	Q	T	T	V	A	S	A	Q	A	N	I	C	L	I	P	E
Leishmania tarentolae	L	M	G	R	E	S	G	F	I	A	A	Q	T	T	V	A	S	A	Q	A	N	I	C	L	I	P	E
Leishmania braziliensis	L	M	G	R	E	S	G	F	I	A	A	Q	T	A	V	A	S	A	Q	A	N	I	C	L	I	P	E
Endotrypanum monterogeii	L	M	G	R	E	S	G	F	I	A	A	Q	T	A	V	A	S	A	Q	A	N	I	C	L	I	P	E

Species	296	297	298	299	300	301	302	303	304	305	306	307	308	309	310	311	312	313	314	315	316	317	318	319	320	321	322
Trypanosoma brucei	N	P	I	S	E	Q	E	V	M	S	L	L	E	R	R	F	C	H	S	R	S	C	V	I	I	V	A
Trypanosoma congolense	N	P	I	S	E	E	E	V	M	A	L	I	E	R	R	F	S	S	S	H	T	C	V	I	I	V	A
Trypanosoma cruzi	N	P	I	S	E	D	I	V	M	A	L	I	Q	R	R	F	E	T	S	R	S	C	V	I	I	V	A
Trypanosoma rangeli	N	P	I	P	E	E	I	V	M	K	L	I	E	R	R	F	A	T	S	R	S	C	V	I	I	V	A
Leishmania infantum	N	P	L	P	K	E	T	V	M	R	L	I	E	R	R	L	Q	Q	S	R	N	C	V	I	V	V	A
Leishmania tarentolae	N	P	L	P	K	E	T	V	M	R	L	I	E	R	R	L	Q	Q	S	C	N	C	V	I	V	V	A
Leishmania braziliensis	N	P	L	P	K	E	T	V	M	R	L	I	E	R	R	F	Q	Q	S	R	N	C	V	I	I	V	A
Endotrypanum monterogeii	N	P	L	P	K	E	T	V	M	R	L	I	E	R	R	L	Q	Q	S	H	S	C	V	V	I	V	A

Species	323	324	325	326	327	328	329	330	331	332	333	334	335	336	337	338	339	340	341	342	343	344	345	346	347	348	349
Trypanosoma brucei	E	G	F	G	Q	D	W	G	R	G	S	G	G	Y	D	A	S	G	N	K	K	L	I	D	I	G	V
Trypanosoma congolense	E	G	F	G	Q	D	W	G	R	G	S	G	G	Y	D	A	S	G	N	K	K	L	I	D	I	G	V
Trypanosoma cruzi	E	G	F	G	Q	D	W	E	G	G	T	G	G	H	D	A	S	G	N	K	K	L	T	D	I	G	V
Trypanosoma rangeli	E	G	F	G	Q	D	W	V	A	D	T	G	G	H	D	A	S	G	N	K	K	L	A	N	I	G	V
Leishmania infantum	E	G	F	G	Q	D	W	E	T	G	T	G	G	H	D	A	S	G	N	K	K	L	V	D	I	G	F
Leishmania tarentolae	E	G	F	G	Q	D	W	E	T	G	T	G	G	H	D	A	S	G	N	K	K	L	V	D	I	G	S
Leishmania braziliensis	E	G	F	G	Q	D	W	E	T	G	V	G	G	H	D	A	S	G	N	K	K	L	I	D	I	G	F
Endotrypanum monterogeii	E	G	F	G	Q	D	W	V	T	S	K	G	G	H	D	A	S	G	N	K	K	L	V	D	I	G	F

Species	350	351	352	353	354	355	356	357	358	359	360	361	362	363	364	365	366	367	368	369	370	371	372	373	374	375	376
Trypanosoma brucei	I	L	T	E	K	V	K	A	F	L	K	A	N	K	S	R	Y	P	D	S	T	V	K	Y	I	D	P
Trypanosoma congolense	I	L	T	K	K	V	N	A	F	L	K	A	N	K	N	R	F	P	D	A	S	V	K	Y	I	D	P
Trypanosoma cruzi	V	L	T	K	R	I	Q	A	W	L	R	K	N	K	E	R	Y	P	N	G	T	V	K	Y	I	D	P
Trypanosoma rangeli	I	L	T	K	R	I	K	A	W	L	A	A	N	K	N	R	F	P	N	G	T	V	K	Y	I	D	P
Leishmania infantum	I	L	K	K	E	V	E	S	W	L	R	A	N	K	E	K	F	P	Q	G	T	V	K	Y	I	D	P
Leishmania tarentolae	I	L	K	K	E	V	E	R	W	L	R	A	N	K	E	K	F	P	Q	G	T	V	K	Y	I	D	P
Leishmania braziliensis	I	L	K	K	E	V	E	S	W	L	R	A	N	K	E	K	Y	P	Q	G	T	V	K	Y	I	D	P
Endotrypanum monterogeii	V	L	K	K	E	V	E	A	W	L	R	S	N	K	A	K	F	P	H	G	T	V	K	Y	I	D	P

Species	377	378	379	380	381	382	383	384	385	386	387	388	389	390	391	392	393	394	395	396	397	398	399	400	401	402	403
Trypanosoma brucei	S	Y	M	I	R	A	C	P	P	S	A	N	D	A	L	F	C	A	T	L	A	T	L	A	V	H	E
Trypanosoma congolense	S	Y	M	I	R	A	C	P	P	S	A	N	D	A	L	F	C	A	T	L	A	T	L	A	V	H	E
Trypanosoma cruzi	S	Y	M	I	R	A	C	P	P	S	A	N	D	A	L	F	C	A	T	L	S	T	L	A	M	H	E
Trypanosoma rangeli	S	Y	M	I	R	A	C	P	P	S	A	N	D	A	L	F	C	A	T	L	A	T	L	A	M	H	E
Leishmania infantum	S	Y	M	I	R	A	C	P	P	S	S	N	D	A	L	F	C	T	N	L	A	T	L	A	V	H	E
Leishmania tarentolae	S	Y	M	I	R	A	C	P	P	S	S	N	D	A	L	F	C	T	T	L	A	T	L	A	V	H	E
Leishmania braziliensis	S	Y	M	I	R	A	C	A	P	S	S	N	D	A	L	F	C	T	T	L	A	T	L	A	V	H	E
Endotrypanum monterogeii	S	Y	M	I	R	A	C	P	P	S	S	N	D	A	L	F	C	T	T	L	A	T	L	A	V	H	E

Species	404	405	406	407	408	409	410	411	412	413	414	415	416	417	418	419	420	421	422	423	424	425	426	427	428	429	430
Trypanosoma brucei	A	M	A	G	A	T	G	C	I	I	A	M	R	H	N	N	Y	I	L	V	P	I	K	V	A	T	S
Trypanosoma congolense	A	M	S	G	A	T	C	C	I	I	A	M	R	Y	N	N	Y	I	L	V	P	I	K	V	A	T	S
Trypanosoma cruzi	A	M	A	G	A	T	N	C	I	I	A	L	R	Y	N	S	Y	I	L	V	P	I	K	V	A	T	S
Trypanosoma rangeli	A	M	A	G	A	T	N	C	I	I	S	M	R	H	N	N	Y	I	L	V	P	I	T	V	A	T	S
Leishmania infantum	A	M	A	G	A	T	G	C	I	I	S	M	R	Y	N	N	Y	I	L	V	P	I	K	A	A	T	S
Leishmania tarentolae	A	M	A	G	A	T	G	C	I	I	S	M	R	Y	N	N	Y	I	L	V	P	I	K	A	A	T	S
Leishmania braziliensis	A	M	A	G	A	T	G	C	I	I	A	L	R	Y	N	N	Y	I	L	V	P	I	K	A	A	T	S
Endotrypanum monterogeii	A	M	A	G	A	T	G	C	I	I	S	L	R	Y	N	N	Y	I	L	V	P	I	K	A	A	T	S

Species	431	432	433	434	435	436	437	438	439	440	441	442	443	444	445	446	447	448	449	450	451	452	453	454	455	456	457
Trypanosoma brucei	V	R	R	V	L	D	L	R	G	Q	L	W	R	Q	V	R	E	I	T	V	D	L	G	S	D	V	R
Trypanosoma congolense	V	R	R	V	L	D	L	R	G	Q	L	W	R	Q	V	R	E	I	T	V	D	L	Q	S	D	V	R
Trypanosoma cruzi	V	R	R	V	L	D	L	R	G	Q	L	W	R	Q	V	R	E	I	T	V	G	L	Q	D	D	V	R
Trypanosoma rangeli	V	R	R	V	L	D	L	R	G	Q	L	W	R	Q	V	R	E	I	T	V	S	L	R	D	N	V	R
Leishmania infantum	V	R	R	V	V	S	L	R	G	A	L	W	R	Q	V	R	E	I	T	V	G	L	S	D	D	V	Q
Leishmania tarentolae	V	R	R	V	V	S	L	R	G	A	L	W	R	Q	V	R	E	I	T	V	G	L	S	D	D	V	H
Leishmania braziliensis	V	R	R	V	V	S	L	R	G	A	L	W	R	Q	V	R	E	I	T	V	G	L	S	D	N	V	Q
Endotrypanum monterogeii	V	R	R	V	V	S	L	R	G	A	L	W	R	Q	V	R	E	I	T	V	G	L	S	D	D	V	S

Species	458	459	460	461	462	463	464	465	466	467	468	469	470	471	472	473	474	475	476	477	478	479	480	481	482	483	484
Trypanosoma brucei	L	A	R	K	L	E	I	R	R	E	L	E	A	I	N	R	N	R	D	R	L	H	E	E	L	A	K
Trypanosoma congolense	A	S	R	R	L	E	I	H	R	E	L	E	A	V	N	R	A	R	E	R	L	M	E	E	M	S	K
Trypanosoma cruzi	A	F	K	E	A	E	V	R	R	E	L	E	A	I	S	L	V	R	E	R	L	I	G	Q	L	S	K
Trypanosoma rangeli	D	C	K	I	T	E	V	R	R	E	L	E	A	I	S	L	S	R	E	R	L	I	E	E	L	S	K
Leishmania infantum	Q	W	N	E	Q	D	L	R	R	H	L	E	S	L	N	V	E	R	E	R	I	I	A	R	L	A	S
Leishmania tarentolae	Q	W	N	E	Q	D	L	R	R	D	L	E	S	L	N	V	E	R	E	R	I	I	A	R	L	A	S
Leishmania braziliensis	Q	W	N	E	Q	D	L	R	R	H	L	E	S	L	N	V	E	R	E	R	I	I	A	R	L	A	S
Endotrypanum monterogeii	Q	W	N	E	Q	D	L	R	R	H	L	D	S	L	N	I	E	R	E	R	I	I	A	R	L	A	S

Species	485	486
Trypanosoma brucei	L	-
Trypanosoma congolense	L	-
Trypanosoma cruzi	L	-
Trypanosoma rangeli	L	-
Leishmania infantum	K	V
Leishmania tarentolae	K	L
Leishmania braziliensis	K	V
Endotrypanum monterogeii	K	V

Figure Supplementary 2 (Figure S2)

Surface Plasmon Resonance sensorgrams for TbPFK, TcPFK, and LiPFK.

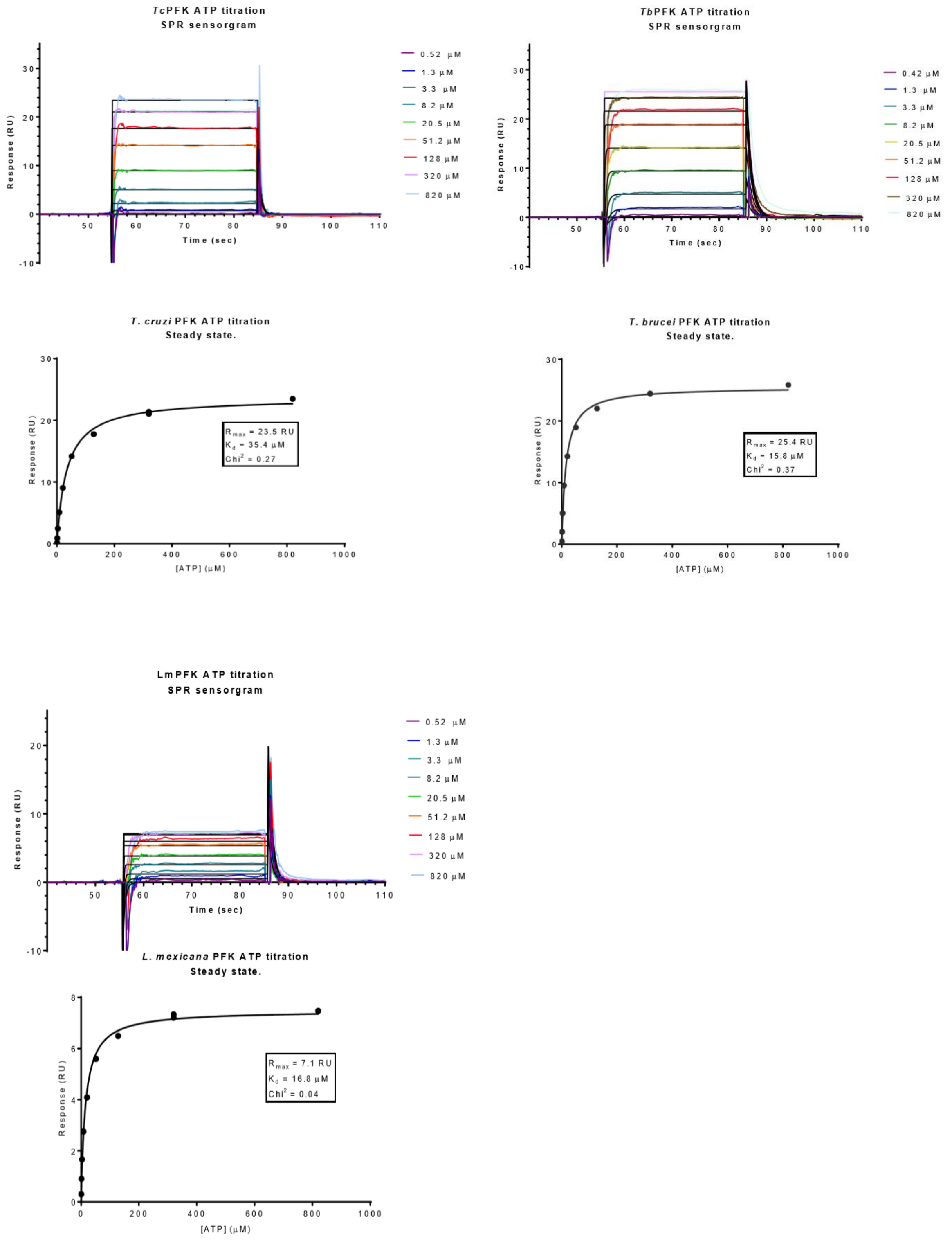

Figure Supplementary 3 (Figure S3)

Thermal denaturation assays of trypanosomatid PFKs. All experiments were carried out in triplicate as described in the methods section, but only a single experiment trace is shown for each PFK.

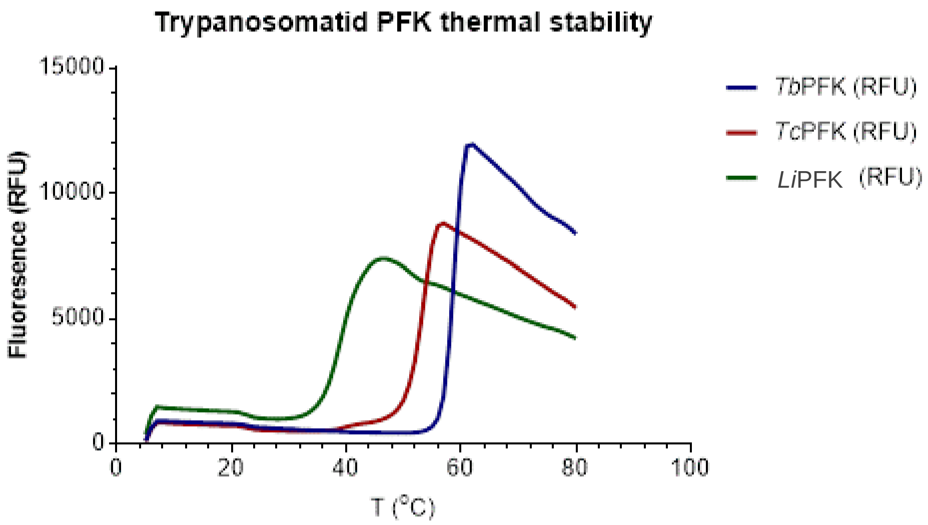

Figure Supplementary 4 (Figure S4)

Melting temperature shifts for trypanosomatid PFKs with substrates. All experiments were carried out in triplicate as described in the methods section. Standard deviation = 0.1-0.3 °C, error bars are not shown.

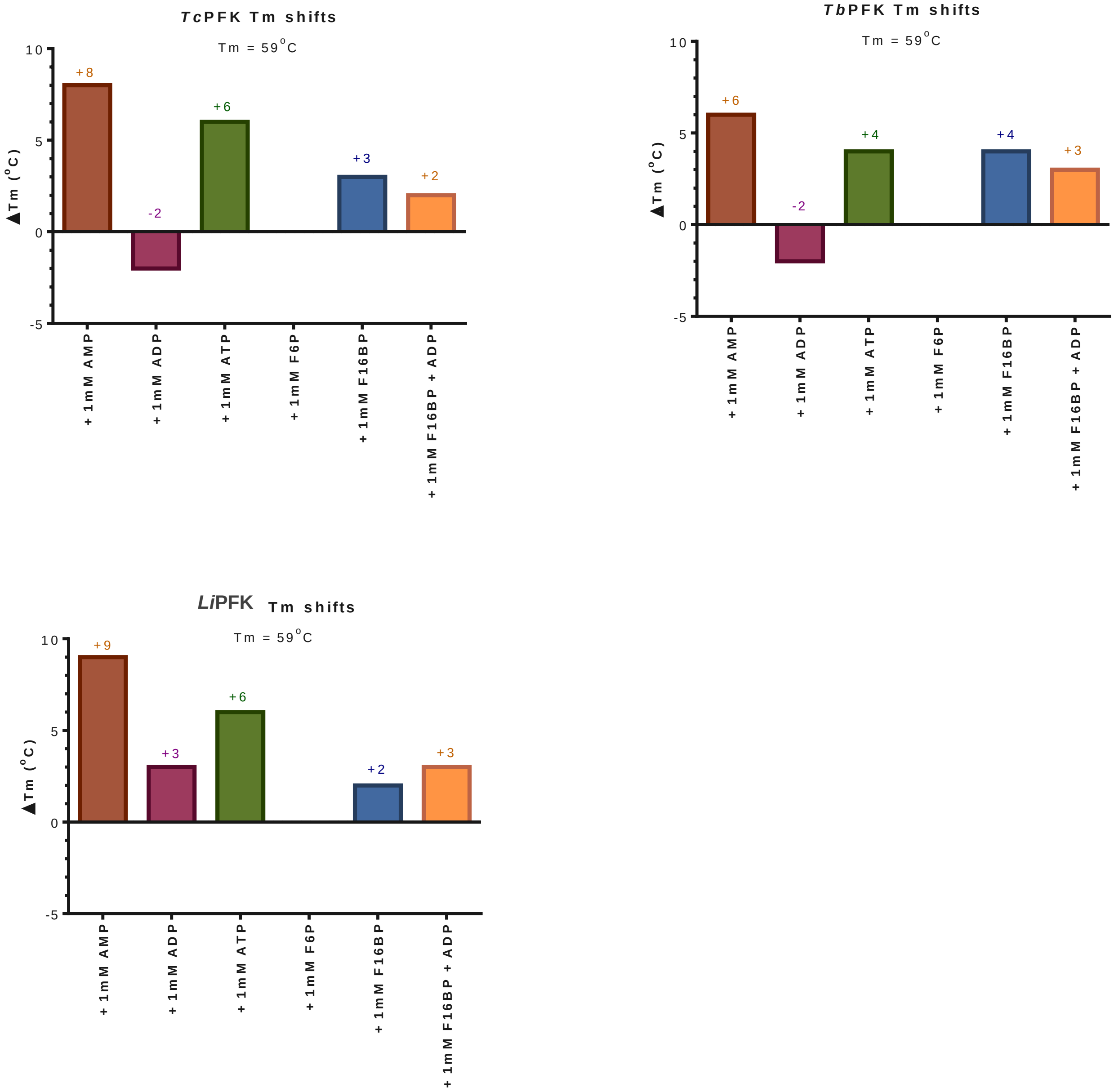

Table Supplementary 1 (Table S1) Crystallographic data for *Trypanosoma brucei* phosphofructokinase (PDB code 6SY7. Values in parentheses are for the highest resolution shell.

Resolution range	39.81 - 2.75 (2.848 - 2.75)
Space group	P 21 21 21
Unit cell	118.395 133.711 271.82 90 90 90
Total reflections	414835 (20616)
Unique reflections	111370 (5467)
Multiplicity	3.8 (3.8)
Completeness (%)	99.0 (99.6)
Mean I/sigma(I)	9.6 (1.5)
R-merge	0.075 (0.604)
R-meas	0.100 (0.808)
R-pim	0.050 (0.398)
CC1/2	0.997 (0.694)
Reflections used in refinement	111279 (11051)
Reflections used for R-free	5586 (549)
R-work	0.1676 (0.2276)
R-free	0.2178 (0.2854)
Number of non-hydrogen atoms	29078
macromolecules	28201
ligands	96
solvent	781
Protein residues	3618
RMS(bonds)	0.011
RMS(angles)	1.50
Ramachandran favored (%)	97.15
Ramachandran allowed (%)	2.04
Ramachandran outliers (%)	0.81
Rotamer outliers (%)	0.03
Clashscore	6.99
Average B-factor	60.83
macromolecules	61.20
ligands	59.57
solvent	47.36
Number of TLS groups	8
